# A unified model-implied instrumental variable approach for structural equation modeling with mixed variables

**DOI:** 10.1007/s11336-021-09771-4

**Published:** 2021-06-07

**Authors:** Shaobo Jin, Fan Yang-Wallentin, Kenneth A. Bollen

**Affiliations:** 1 Department of statistics, Uppsala University, Uppsala, Sweden; 2 Department of psychology and neuroscience, Department of sociology, University of North Carolina at Chapel Hill, Chapel Hill, USA

**Keywords:** MIIV, continuous variables, ordinal variables, goodness-of-fit test, overidentification test

## Abstract

The model-implied instrumental variable (MIIV) estimator is an equation-by-equation estimator of structural equation models that is more robust to structural misspecifications than full information estimators. Previous studies have concentrated on endogenous variables that are all continuous (MIIV-2SLS) or all ordinal . We develop a unified MIIV approach that applies to a mixture of binary, ordinal, censored, or continuous endogenous observed variables. We include estimates of factor loadings, regression coefficients, variances, and covariances along with their asymptotic standard errors. In addition, we create new goodness of fit tests of the model and overidentification tests of single equations. Our simulation study shows that the proposed MIIV approach is more robust to structural misspecifications than diagonally weighted least squares (DWLS) and that both the goodness of fit model tests and the overidentification equations tests can detect structural misspecifications. We also find that the bias in asymptotic standard errors for the MIIV estimators of factor loadings and regression coefficients are often lower than the DWLS ones, though the differences are small in large samples. Our analysis shows that scaling indicators with low reliability can adversely affect the MIIV estimators. Also, using a small subset of MIIVs reduces small sample bias of coefficient estimates, but can lower the power of overidentification tests of equations.

**Supplementary Information:**

The online version contains supplementary material available at 10.1007/s11336-021-09771-4.

## Introduction

Structural equation modeling (SEM) is widely used in social and behavior sciences. Endogenous observed variables (e.g., indicators) in a SEM model can be continuous, binary, ordinal, or censored. When all endogenous observed variables are continuous, systemwide maximum likelihood (ML) is the dominant estimator. When binary or ordinal observed endogenous variables are present, researchers frequently use unweighted least squares (ULS, Muthén, 1978) and diagonally weighted least squares (DWLS, Muthén et al., 1997). Typically, these estimators require the estimation of a polychoric correlation matrix as a first stage. In the second stage, they use the polychoric correlation matrix as input and apply systemwide estimators such as ML, ULS, and DWLS. They are systemwide in the sense that all parameter estimates for all equations in the whole structural equation model are estimated simultaneously. Despite their popularity, various studies (e.g., Bollen, 1996; Jin et al., 2016; Nestler, 2013; Yang-Wallentin et al., 2010) have shown that the systemwide estimators can spread the bias due to model misspecifications such as nonzero coefficients (“omitted variables") or covariances of errors mistakenly set to zero. Our focus is on these types of misspecifications.

To reduce the impacts of such model misspecifications, Bollen (1996) proposed a two-stage least squares (2SLS) approach using model-implied instrumental variables (MIIVs) when all endogenous observed variables are continuous. Bollen and Maydeu-Olivares (2007) extend this approach to models with observed ordinal endogenous variables and called it the polychoric instrumental variable (PIV) estimator. The MIIVs for the 2SLS and PIV estimators are easily found from the pool of variables in the SEM model by the algorithm described in Bollen (1996, page 114) and implemented in SAS by Bollen and Bauer (2004), in Stata by Bauldry (2014), and in R by Fisher et al. (2017). The instrumental variables from this approach are model-implied instrumental variables; in that according to the structure of the model, these MIIVs should be uncorrelated with the error of the equation to be estimated. 2SLS with MIIVs is denoted by MIIV-2SLS to distinguish it from the 2SLS with auxiliary, external instrumental variables that are not part of the original model. In practice, auxiliary instruments (Bollen, 2012) are more common than MIIVs so it is important to distinguish between them. Both MIIV-2SLS and PIV approaches estimate the parameters in an equation-by-equation manner, in which each indicator equation with its factor loadings and each latent variable equation with its regression coefficients is estimated separately from each other. Furthermore, the estimator is non-interative (Bollen, 1996), which eliminates the non-convergence issue that can occur with the systemwide estimators. The simulation study of Bollen et al. (2007) showed that the MIIV-2SLS estimator has accuracy comparable to the traditional ML estimator if the model is correctly specified and is often more robust if the model is misspecified. Similarly, Jin et al. (2016) and Nestler (2013) showed that the PIV estimator is as accurate as the ULS and DWLS estimators in the correctly specified model and is more accurate in the structurally misspecified model. The reader is directed to Bollen et al. (2018a) and Bollen (2020) for conditions under which the MIIV-2SLS and PIV approaches remain robust to structural misspecifications. Recently, the principle of MIIV-2SLS and PIV has been generalized by Bollen et al. (2014) to SEM with generalized method of moments estimators, by Fisher et al. (2019) to dynamic time-series models, by Nestler (2014) to handle equality constraints, by Nestler (2015a) to nonlinear SEM models, and by Nestler (2015b) to growth curve models, just to name a few.

Another advantage of the MIIV-2SLS and PIV method is the local, equation-specific test of model specification. The most common method to improve model fit and search for model misspecification with systemwide estimators is to sequentially apply the modification index (Sörbom, 1989). However, the modification index can lead to severe errors (MacCallum, 1986; MacCallum et al., 1992). Bollen (1996, pages 117-118) and Kirby and Bollen (2009) recommend the Sargan (Sargan, 1958) test for equation-by-equation overidentification tests of misspecification. Jin and Cao (2018) showed that these tests are not suitable for ordinal endogenous variables and proposed alternative overidentification tests for such variables.

The cited studies focused on situations where all endogenous variables were either continuous (MIIV-2SLS) or all were ordinal (PIV). To our knowledge, the only exception is a parallel study by Fisher and Bollen (2020), which also considered inference using MIIV with different types of observed variables. The main purpose of our study is to unify the MIIV-2SLS and PIV approaches and propose an approach for a SEM model with different variable types, such as continuous, ordinal, binary, or a mixture of different types. Hereafter, the unified approach is termed the MIIV approach. We will present the MIIV point estimators, which is in line with the estimator in Fisher and Bollen (2020). Two sets of standard errors are proposed. One set of standard errors matches those in Bollen (1996) for observed continuous variables in finite sample and is asymptotically equivalent to those in Bollen and Maydeu-Olivares (2007) for observed ordinal variables. The other is asymptotically equivalent to Bollen (1996) but equivalent to Bollen and Maydeu-Olivares (2007) in finite samples. Fisher and Bollen (2020) only focused on point estimation and standard error estimation. In contrast, we will also develop goodness-of-fit tests for the whole model and overidentification tests for individual equations. Similarly to the standard error estimators, one set of overidentification tests for equations matches Kirby and Bollen (2009) for observed continuous variables and the other set matches Jin and Cao (2018) for observed ordinal variables.

The rest of the paper is organized as follows. We first briefly present the SEM model and the MIIV idea. Then, we develop the estimation of SEMs with the unified MIIV approach. Next, we provide model goodness-of-fit tests and overidentification tests of equations. We conduct a Monte Carlo simulation to investigate the small sample properties of the proposed approach. An empirical example illustrates our approach. A discussion and conclusion section ends the paper.

## Structural Equation Modeling and Instrumental Variable

### SEM Model

Consider the SEM model

1 $$\begin{aligned} {\varvec{y}}^{*}= & {} {\varvec{\Lambda }} {\varvec{\eta }}+{\varvec{\varepsilon }}, \end{aligned}$$

2 $$\begin{aligned} {\varvec{\eta }}= & {} {\varvec{B}} {\varvec{\eta }} +{\varvec{\zeta }}, \end{aligned}$$

where ${\varvec{y}}^{*}$ is the vector of continuous variables, ${\varvec{\Lambda }}$ is the matrix of factor loadings, ${\varvec{\eta }}$ is the vector of latent variables, ${\varvec{B}}$ is the matrix of latent regression coefficient, and ${\varvec{\varepsilon }}$ and ${\varvec{\zeta }}$ are disturbances. Without loss of generality, we assume that ${\varvec{y}}^{*}$ has a zero mean. We can justify the zero mean assumption by forming mean deviation variables for the observed continuous variables and for observed binary and ordinal variables researchers commonly assume that their underlying variables have means of zero. We also assume that ${\varvec{\eta }}$ and ${\varvec{\varepsilon }}$ are independent and that ${\varvec{\zeta }}$ and ${\varvec{\varepsilon }}$ are independent. The ${\varvec{\eta }}$ vector consists of endogenous and exogenous latent variables with the exogenous variables independent of the errors in ${\varvec{\zeta }}$ that correspond to the endogenous variables. Furthermore, the disturbances ${\varvec{\varepsilon }}$ and ${\varvec{\zeta }}$ are homoscedastic, where $\text {var}({\varvec{\varepsilon }}) = {\varvec{\Theta }}$ and $\text {var}({\varvec{\zeta }}) = {\varvec{\Psi }}$.

The observed vector is denoted by ${\varvec{y}}$, which is not necessarily the same as ${\varvec{y}}^{*}$. If $y_j$, the *j*th entry in ${\varvec{y}}$, is continuous, then $y_j = y_j^*$, where $y_j^*$ is the *j*th entry in ${\varvec{y}}^*$. If $y_j$ is binary or ordinal, then it is obtained by categorizing the underlying continuous variable $y_j^*$ according to the threshold values. If $y_j$ is censored, then it is obtained by censoring $y_j^*$ at a boundary. If ${\varvec{y}}^{*}$ only contains observed continuous variables, we only need it to have finite moments up to the fourth order. If ${\varvec{y}}^{*}$ contains any observed binary, ordinal, or censored variables, we assume ${\varvec{\eta }}$, ${\varvec{\varepsilon }}$, and ${\varvec{\zeta }}$ follow a multivariate normal distribution.

Let ${\varvec{\Sigma }}$ be the population covariance matrix of ${\varvec{y}}^{*}$. If some entries in ${\varvec{y}}^{*}$ are discrete, the corresponding variances are fixed to 1 for identification. Estimation of ${\varvec{S}}$, the sample counterpart of ${\varvec{\Sigma }}$, depends on the types of indicators. For example, using Olsson (1979), we can estimate the polychoric correlation for two ordinal variables, and using Olsson et al. (1982), we can estimate the polyserial correlation for one ordinal indicator and one continuous indicator. Throughout the paper, we assume that ${\varvec{S}}$ is a consistent estimator of ${\varvec{\Sigma }}$ and that

$$\begin{aligned} \sqrt{n}\left( {\varvec{s}}-{\varvec{\sigma }}\right)&\overset{d}{\rightarrow }&N\left( {\varvec{0}},{\varvec{\Upsilon }}\right) , \end{aligned}$$

where *n* is the sample size, ${\varvec{s}}$ is the vector of nonredundant free entries in ${\varvec{S}}$, ${\varvec{\sigma }}$ is the vector of corresponding free entries in ${\varvec{\Sigma }}$, and ${\varvec{\Upsilon }}$ is the asymptotic covariance matrix.

Our SEM model implies that ${\varvec{\Sigma }}$ equals the implied covariance matrix of

3 $$\begin{aligned} {\varvec{\Sigma }} \left( {\varvec{\theta }} \right)= & {} {\varvec{\Lambda }} \left( {\varvec{I}}-{\varvec{B}} \right) ^{-1} {\varvec{\Psi }} \left( {\varvec{I}}-{\varvec{B}} \right) ^{-\mathrm{T}} {\varvec{\Lambda }}^{\mathrm{T}}+{\varvec{\Theta }}, \end{aligned}$$

where ${\varvec{\theta }}$ is the vector of all model parameters (i.e., free parameters in ${\varvec{\Lambda }}$, ${\varvec{B}}$, ${\varvec{\Psi }}$, and ${\varvec{\Theta }}$) and $(\cdot )^{-\mathrm{T}}$ takes the transpose of the inverse of the enclosed matrix. The diagonal entries of ${\varvec{\Theta }}$ that correspond to binary and ordinal indicators are not free parameters, but are restricted so that the corresponding entries in ${\varvec{\Sigma }} \left( {\varvec{\theta }} \right)$ are 1 for identification. The traditional systemwide approaches estimate ${\varvec{\theta }}$ by minimizing the fit function

4 $$\begin{aligned} T\left( {\varvec{\theta }} \right)= & {} \left( {\varvec{s}}-{\varvec{\sigma }}({\varvec{\theta }})\right) ^{T}\hat{{\varvec{W}}} \left( {\varvec{s}}-{\varvec{\sigma }}({\varvec{\theta }})\right) , \end{aligned}$$

where ${\varvec{\sigma }}({\varvec{\theta }})$ is the vector of free entries in ${\varvec{\Sigma }} \left( {\varvec{\theta }} \right)$ and the weight matrix $\hat{{\varvec{W}}}$ is a consistent estimator of ${\varvec{W}}$. Different weight matrices yield different estimators, e.g., ${\varvec{W}}={\varvec{I}}$ in unweighted least squares (ULS; Muthén, 1978), ${\varvec{W}}$ is the inverse of diagonal elements of ${\varvec{\Upsilon }}$ in diagonally weighted least squares (DWLS; Muthén et al., 1997), and ${\varvec{W}}={\varvec{\Upsilon }}^{-1}$ in weighted least squares (WLS; Browne, 1984).

### Latent to Observed Variable (L2O) Transformation

Each latent variable must be given a scale or metric. To set the scale of ${\varvec{\eta }}$, the most common approach is to choose one indicator per latent variable and to set its factor loading to one so that it becomes the scaling indicator. When possible, we should choose the scaling indicator to have factor complexity of one and a high $R^2$. Suppose that we partition ${\varvec{y}}^{*}$ into $( {\varvec{y}}^{*T}_1, {\varvec{y}}^{*T}_2 )^T$ and ${\varvec{\varepsilon }}$ into $( {\varvec{\varepsilon }}_1^T, {\varvec{\varepsilon }}_2^T )^T$ such that ${\varvec{y}}^{*}_{1} = {\varvec{\eta }} + {\varvec{\varepsilon }}_{1}$ is for the scaling indicators and ${\varvec{y}}^{*}_{2} = {\varvec{\Lambda }}_2 {\varvec{\eta }} + {\varvec{\varepsilon }}_{2}$ contains unknown factor loadings for the nonscaling indicators. Following Bollen and Maydeu-Olivares (2007), the SEM model is equivalent to

5 $$\begin{aligned} \begin{pmatrix}{\varvec{y}}^{*}_{2}\\ {\varvec{y}}^{*}_{1} \end{pmatrix}= \begin{pmatrix} {\varvec{\Lambda }}_{2} \\ {\varvec{B}}\\ \end{pmatrix}{\varvec{y}}^{*}_{1}+\begin{pmatrix} {\varvec{\varepsilon }}_{2}-{\varvec{\Lambda }}_{2}{\varvec{\varepsilon }}_{1} \\ {\varvec{\zeta }}+\left( {\varvec{I}}-{\varvec{B}}\right) {\varvec{\varepsilon }}_{1} \end{pmatrix}, \end{aligned}$$

which is simply a multivariate regression system with “observed” variables. This is referred to as the Latent-to-Observed (L2O) variable transformation (Bollen, 2019), because the original equation system with latent variables is transformed to one without latent variables. When we have noncontinuous endogenous observed variables, the L2O transformation results in underlying scaling variables rather than observed ones. Regardless, in most cases, this leads the regressors to be correlated with one or more parts of the composite error terms. We can use MIIVs to consistently estimate the regression coefficients in ${\varvec{B}}$ and ${\varvec{\Lambda }}_2$.

Equation (5) indicates that we can partition ${\varvec{\theta }}$ into two vectors: ${\varvec{\theta }}_1$ (free parameters in ${\varvec{\Lambda }}_2$ and ${\varvec{B}}$) and ${\varvec{\theta }}_2$ (the nonredundant free parameters in ${\varvec{\Psi }}$ and ${\varvec{\Theta }}$). To estimate ${\varvec{\theta }}_1$, Bollen (1996) and Bollen and Maydeu-Olivares (2007) proposed to consider the equation system (5) in a row-by-row manner. Suppose that the *j*th row of the system (5) is

6 $$\begin{aligned} y_{j}^*= & {} {\varvec{z}}_{j}^{*T} {\varvec{\theta }}_1^{(j)}+e_{j}, \end{aligned}$$

where ${\varvec{z}}_{j}^*$ is the right-hand side explanatory variable vector, and ${\varvec{\theta }}_1^{(j)}$ is the vector of parameters. We assume that there exists a non-empty subset of ${\varvec{z}}_{j}^*$ that is correlated with the error $e_{j}$. As the names implies, the MIIVs, denoted by ${\varvec{v}}_{j}^*$, are selected from the variables ${\varvec{y}}^{*}$, excluding $y_{j}^*$ and the elements in ${\varvec{z}}_{j}^*$ that have nonzero correlations with $e_{j}$. The valid MIIVs ${\varvec{v}}_{j}^*$ must be correlated with the endogenous variable ${\varvec{z}}_{j}^*$ as well as uncorrelated with the error term $e_{j}$, and the number of ${\varvec{v}}_{j}^*$ must be no lower than the number of ${\varvec{z}}_{j}^*$. We can find the pool of all valid MIIVs for a given equation by using the algorithms proposed by Bollen (1996). Due to space limitation, we direct the readers to Bollen (2019) for examples of Eq. (6) and the algorithm of selecting valid IVs. It is worth mentioning that the choice of the scaling indicator will affect the choice of MIIVs and the estimates. Though the estimator remains consistent, its asymptotic variance could differ. As mentioned earlier, it is preferable to choose as scaling indicators those indicators that are thought to be most closely related to the latent variable that they measure.

Bollen (1996; 2001), Bollen et al. (2007), and Kirby and Bollen (2009) have investigated the case where ${\varvec{y}}$ is continuous. In contrast, Bollen and Maydeu-Olivares (2007), Jin et al. (2016), Jin and Cao (2018), and Nestler (2013) have investigated the case where ${\varvec{y}}$ is ordinal. Fisher and Bollen (2020) is the only study we know of that considered indicators of different types.

## Estimation by the MIIV Approach

In this section, we propose a unified approach for estimating and testing models with continuous, binary, or ordinal endogenous observed variables. The approach applies as long as the sample moment matrix is a consistent estimator of the population moment matrix, with an estimate of the asymptotic covariance matrix of the elements of such matrices.

### Point Estimator of $\mathbf {\theta }_1$

If Eq. (6) is correctly specified and the MIIVs are valid, then ${\varvec{\theta }}_1^{(j)} = {\varvec{\gamma }}_{j}\left( {\varvec{\sigma }}\right)$, where

$$\begin{aligned} {\varvec{\gamma }}_{j}\left( {\varvec{\sigma }}\right)= & {} \left( {\varvec{\Sigma }}_{vz,j}^{T}{\varvec{\Sigma }}_{vv,j}^{-1}{\varvec{\Sigma }}_{vz,j}\right) ^{-1}{\varvec{\Sigma }}_{vz,j}^{T}{\varvec{\Sigma }}_{vv,j}^{-1}{\varvec{\Sigma }}_{vy,j} , \end{aligned}$$

with ${\varvec{\Sigma }}_{vz,j}$ being the covariance matrix between ${\varvec{v}}_{j}^*$ and ${\varvec{z}}_{j}^*$, and ${\varvec{\Sigma }}_{vv,j}$ being the covariance matrix of ${\varvec{v}}_{j}^*$, and ${\varvec{\Sigma }}_{vy,j}$ being the covariance matrix between ${\varvec{v}}^*_{j}$ and $y^*_{j}$. The MIIV estimator is

7 $$\begin{aligned} \hat{{\varvec{\theta }}}_1^{(j)} = {\varvec{\gamma }}_{j}\left( {\varvec{s}}\right) =\left( {\varvec{S}}_{vz,j}^{T}{\varvec{S}}_{vv,j}^{-1}{\varvec{S}}_{vz,j}\right) ^{-1}{\varvec{S}}_{vz,j}^{T}{\varvec{S}}_{vv,j}^{-1}{\varvec{S}}_{vy,j}, \end{aligned}$$

where ${\varvec{S}}_{\cdot \cdot ,j}$ is the sample counterpart of ${\varvec{\Sigma }}_{\cdot \cdot ,j}$. The same estimator was proposed by Bollen and Maydeu-Olivares (2007) and Fisher and Bollen (2020). If all observed variables are continuous, the MIIV estimator (7) is equivalent to the estimator in Bollen (1996)

$$\begin{aligned} \hat{{\varvec{\theta }}}_1^{(j)}=\left[ {\varvec{Z}}_{j}^{T}{\varvec{V}}_{j}\left( {\varvec{V}}_{j}^{T}{\varvec{V}}_{j}\right) ^{-1}{\varvec{V}}_{j}^{T}{\varvec{Z}}_{j}\right] ^{-1}{\varvec{Z}}_{j}^{T}{\varvec{V}}_{j}\left( {\varvec{V}}_{j}^{T}{\varvec{V}}_{j}\right) ^{-1}{\varvec{V}}_{j}^{T}{\varvec{y}}_{j}, \end{aligned}$$

where ${\varvec{y}}_{j}$, ${\varvec{Z}}_{j}$, and ${\varvec{V}}_{j}$ are the demeaned data matrices of $y^*_{j}$, ${\varvec{z}}^*_{j}$, and ${\varvec{v}}^*_{j}$, respectively. Similar to MIIV-2SLS and PIV, the MIIV estimator is a consistent estimator of ${\varvec{\theta }}_{1}^{(j)}$, as long as Eq. (6) is correctly specified, the inverses in Eq. (7) exist, and the MIIVs are valid. This implies that we can maintain consistency of $\hat{{\varvec{\theta }}}_1^{(j)}$ in a misspecified SEM model under certain conditions (e.g., Bollen, 2001; Bollen et al., 2018b; Bollen, 2020). In contrast, the systemwide estimators sometimes spread the bias from a poorly specified equation in one part of the model to a correctly specified equation in another part.

### Standard Error of $\varvec{{\hat{\mathbf { \theta } }} }_{1}$

If we collect $\hat{{\varvec{\theta }}}_1^{(j)}$ for all *j*, the MIIV estimator of ${\varvec{\theta }}_1$ is $\hat{{\varvec{\theta }}}_1 = {\varvec{\gamma }} \left( {\varvec{s}}\right)$, where ${\varvec{\gamma }} \left( {\varvec{\sigma }}\right)$ is the vector that stacks all ${\varvec{\gamma }}_{j}\left( {\varvec{\sigma }}\right)$ on top of the other. The delta method indicates that

8 $$\begin{aligned} \sqrt{n}\left( \hat{{\varvec{\theta }}}_{1}-{\varvec{\theta }}_{1}^{*}\right)= & {} {\varvec{K}}\left( {\varvec{\sigma }}\right) \sqrt{n}\left( {\varvec{s}}-{\varvec{\sigma }}\right) +o_{\text {P}}\left( 1\right) , \end{aligned}$$

where ${\varvec{\theta }}_1^{*}$ is the asymptotic limit of $\hat{{\varvec{\theta }}}_1$ and ${\varvec{K}}\left( {\varvec{\sigma }}\right) =\partial {\varvec{\gamma }}\left( {\varvec{\sigma }}\right) /\partial {\varvec{\sigma }}^{T}$. Then, we obtain

9 $$\begin{aligned} \sqrt{n}\left( \hat{{\varvec{\theta }}}_{1}^{(j)}-{\varvec{\theta }}_{1}^{(j)*}\right)&\overset{d}{\rightarrow }&N\left( {\varvec{0}},{\varvec{K}}_{j}\left( {\varvec{\sigma }}\right) {\varvec{\Upsilon }}{\varvec{K}}_{j}^{T}\left( {\varvec{\sigma }}\right) \right) , \end{aligned}$$

where ${\varvec{\theta }}_1^{(j)*}$ is the asymptotic limit of $\hat{{\varvec{\theta }}}_1^{(j)}$ and ${\varvec{K}}_{j}\left( {\varvec{\sigma }}\right) =\partial {\varvec{\gamma }}_{j}\left( {\varvec{\sigma }}\right) /\partial {\varvec{\sigma }}^{T}$. The asymptotic distribution (9) holds even when the MIIVs are invalid and ${\varvec{\theta }}_1^{(j)*}$ is not the same as ${\varvec{\theta }}_1^{(j)}$. To compute ${\varvec{K}}_{j}\left( {\varvec{\sigma }}\right)$, the chain rule yields

10 $$\begin{aligned} \frac{\partial {\varvec{\gamma }}_{j}\left( {\varvec{\sigma }}\right) }{\partial \sigma _{i}}= & {} \left( {\varvec{\Sigma }}_{vz,j}^{T}{\varvec{\Sigma }}_{vv,j}^{-1}{\varvec{\Sigma }}_{vz,j}\right) ^{-1}\frac{\partial {\varvec{\Sigma }}_{vz,j}^{T}{\varvec{\Sigma }}_{vv,j}^{-1}}{\partial \sigma _{i}}\left[ {\varvec{\Sigma }}_{vy,j}-{\varvec{\Sigma }}_{vz,j}{\varvec{\gamma }}_{j}\left( {\varvec{\sigma }}\right) \right] \nonumber \\&+\left( {\varvec{\Sigma }}_{vz,j}^{T}{\varvec{\Sigma }}_{vv,j}^{-1}{\varvec{\Sigma }}_{vz,j}\right) ^{-1}{\varvec{\Sigma }}_{vz,j}^{T}{\varvec{\Sigma }}_{vv,j}^{-1}\left[ \frac{\partial {\varvec{\Sigma }}_{vy,j}}{\partial \sigma _{i}}-\frac{\partial {\varvec{\Sigma }}_{vz,j}}{\partial \sigma _{i}}{\varvec{\gamma }}_{j}\left( {\varvec{\sigma }}\right) \right] , \end{aligned}$$

where $\sigma _i$ is the *i*th entry in ${\varvec{\sigma }}$.

To obtain the estimator of the standard error, ${\varvec{K}}_{j}\left( {\varvec{\sigma }}\right)$ is estimated by ${\varvec{K}}_{j}\left( {\varvec{s}}\right)$ and estimation of ${\varvec{\Upsilon }}$ depends on the distributional assumption. In general, we can estimate ${\varvec{\Upsilon }}$ using the approach in Muthén (1984). If all observed variables are ordinal, we can estimate ${\varvec{\Upsilon }}$ from Jöreskog (1994). If all observed variables are continuous, we estimate ${\varvec{\Upsilon }}$ using the fourth-order moments as in the asymptotic distribution-free approach (Browne, 1984). If all observed variables are normally distributed, we can estimate ${\varvec{\Upsilon }}$ by $2{\varvec{D}}^{+}\left( {\varvec{S}}\otimes {\varvec{S}}\right) {\varvec{D}}^{+T}$, where ${\varvec{D}}$ is a duplication matrix and ${\varvec{D}}^{+}=\left( {\varvec{D}}^{T}{\varvec{D}}\right) ^{-1}{\varvec{D}}^{T}$ (Browne, 1987).

### Equivalence of Standard Errors of $\varvec{{\hat{\mathbf { \theta } }} }_{1}$

The asymptotic distribution attained by Bollen and Maydeu-Olivares (2007) when all observed variables are ordinal is the same as the asymptotic distribution (9). The same distribution is also used by Fisher and Bollen (2020). As mentioned above, this asymptotic distribution is applicable regardless of the validity of the MIIVs. If Eq. (6) is correctly specified and all MIIVs are valid, ${\varvec{\Sigma }}_{vy,j}={\varvec{\Sigma }}_{vz,j}{\varvec{\theta }}_{1}^{(j)}$ and ${\varvec{\gamma }}_{j}\left( {\varvec{\sigma }}\right) ={\varvec{\theta }}_{1}^{(j)}$, then the first term in equation (10) vanishes and ${\varvec{K}}_j\left( {\varvec{\sigma }}\right) {\varvec{\Upsilon }}{\varvec{K}}_j^{T}\left( {\varvec{\sigma }}\right)$ reduces to

11 $$\begin{aligned} \left( {\varvec{\Sigma }}_{vz,j}^{T}{\varvec{\Sigma }}_{vv,j}^{-1}{\varvec{\Sigma }}_{vz,j}\right) ^{-1}{\varvec{\Sigma }}_{vz,j}^{T}{\varvec{\Sigma }}_{vv,j}^{-1} {\varvec{\Omega }}_j {\varvec{\Sigma }}_{vv,j}^{-1}{\varvec{\Sigma }}_{vz,j}\left( {\varvec{\Sigma }}_{vz,j}^{T}{\varvec{\Sigma }}_{vv,j}^{-1}{\varvec{\Sigma }}_{vz,j}\right) ^{-1}&, \end{aligned}$$

where $${\varvec{g}}_{j}\left( {\varvec{\sigma }}\right) ={\varvec{\Sigma }}_{vy,j}-{\varvec{\Sigma }}_{vz,j}{\varvec{\theta }}_{1}^{(j)}$$ and

$$\begin{aligned} {\varvec{\Omega }}_{j}=&\left( \frac{\partial {\varvec{g}}_{j}\left( {\varvec{\sigma }}\right) }{\partial {\varvec{\sigma }}^{T}}\right) {\varvec{\Upsilon }}\left( \frac{\partial {\varvec{g}}_{j}\left( {\varvec{\sigma }}\right) }{\partial {\varvec{\sigma }}^{T}}\right) ^{T}. \end{aligned}$$

The covariance matrix (11) can be estimated by

12 $$\begin{aligned} \left( {\varvec{S}}_{vz,j}^{T}{\varvec{S}}_{vv,j}^{-1}{\varvec{S}}_{vz,j}\right) ^{-1}{\varvec{S}}_{vz,j}^{T}{\varvec{S}}_{vv,j}^{-1} \hat{{\varvec{\Omega }}}_j {\varvec{S}}_{vv,j}^{-1}{\varvec{S}}_{vz,j}\left( {\varvec{S}}_{vz,j}^{T}{\varvec{S}}_{vv,j}^{-1}{\varvec{S}}_{vz,j}\right) ^{-1}, \end{aligned}$$

where $\hat{{\varvec{\Omega }}}_j$ is the estimator of ${\varvec{\Omega }}$ with ${\varvec{\Sigma }}$ evaluated at ${\varvec{S}}$ and ${\varvec{\Upsilon }}$ evaluated at some estimator $\hat{{\varvec{\Upsilon }}}$.

If all observed variables are continuous and all MIIVs are valid, Bollen (1996) showed that

13 $$\begin{aligned} \sqrt{n}\left( \hat{{\varvec{\theta }}}_{1}^{(j)}-{\varvec{\theta }}_{1}^{(j)}\right) \overset{d}{\rightarrow }&N\left( {\varvec{0}},\varphi _{j}^{2}{\varvec{\Sigma }}_{{\hat{z}}{\hat{z}},j}^{-1}\right) , \end{aligned}$$

where ${\varvec{\Sigma }}_{{\hat{z}}{\hat{z}},j}={\varvec{\Sigma }}_{vz,j}^{T}{\varvec{\Sigma }}_{vv,j}^{-1}{\varvec{\Sigma }}_{vz,j}$ is the asymptotic covariance matrix of the predicted right-hand side endogenous variable of Eq. (6) and $\varphi _{j}^{2} = {\varvec{\Sigma }}_{yy,j}-2{\varvec{\theta }}^{(j)T}{\varvec{\Sigma }}_{zy,j}+{\varvec{\theta }}^{(j)T}{\varvec{\Sigma }}_{zz,j}{\varvec{\theta }}^{(j)}$ is the variance of the error term $e_{j}$. We expect the asymptotic covariance matrix $\varphi _{j}^{2}{\varvec{\Sigma }}_{{\hat{z}}{\hat{z}},j}^{-1}$ to be equivalent to (11) when all observed variables are continuous and all MIIVs are valid, since both are asymptotic covariance matrices of $\hat{{\varvec{\theta }}}_{1}^{(j)}$. It is worth emphasizing that we need all MIIVs to be valid in order for (11) and (13) to be valid, but neither the observed continuous variables nor the disturbances need to be normally distributed (Greene, 2002, page 77). We estimate the asymptotic covariance matrix $\varphi _{j}^{2}{\varvec{\Sigma }}_{{\hat{z}}{\hat{z}},j}^{-1}$ by ${\hat{\varphi }}_{j}^{2}\left( {\varvec{S}}_{vz,j}^{T}{\varvec{S}}_{vv,j}^{-1}{\varvec{S}}_{vz,j}\right) ^{-1}$, where ${\hat{\varphi }}_{j}^{2}={\varvec{S}}_{yy,j}-2\hat{{\varvec{\theta }}}_{1}^{(j)T}{\varvec{S}}_{zy,j}+\hat{{\varvec{\theta }}}_{1}^{(j)T}{\varvec{S}}_{zz,j}\hat{{\varvec{\theta }}}_{1}^{(j)}$.

Despite the equivalence of $\varphi _{j}^{2}{\varvec{\Sigma }}_{{\hat{z}}{\hat{z}},j}^{-1}$ and equation (11), their finite sample estimators may still differ, as revealed in the following proposition. For ease of presentation, all proofs are placed in the appendix.

#### Proposition 1

Suppose that all observed variables are continuous and ${\varvec{\Upsilon }}$ is estimated by $2{\varvec{D}}^{+}\left( {\varvec{S}}\otimes {\varvec{S}}\right) {\varvec{D}}^{+T}$. Then,

$$\begin{aligned} \hat{{\varvec{\Omega }}}_j = {\hat{\varphi }}_{j}^{2}{\varvec{S}}_{vv,j}+\left( {\varvec{S}}_{vy,j}-{\varvec{S}}_{vz,j}\hat{{\varvec{\theta }}}_1^{(j)}\right) \left( {\varvec{S}}_{vy,j}-{\varvec{S}}_{vz,j}\hat{{\varvec{\theta }}}_1^{(j)}\right) ^{T}, \end{aligned}$$

and the estimator (12) is the same as ${\hat{\varphi }}_{j}^{2}\left( {\varvec{S}}_{vz,j}^{T}{\varvec{S}}_{vv,j}^{-1}{\varvec{S}}_{vz,j}\right) ^{-1}$.

Proposition 1 shows that the estimator $2{\varvec{D}}^{+}\left( {\varvec{S}}\otimes {\varvec{S}}\right) {\varvec{D}}^{+T}$ equates the estimator (12) and the standard error in Bollen (1996) in finite samples. Such an estimator is a consistent estimator of ${\varvec{\Upsilon }}$ under the normality assumption. In contrast, if the normality assumption is relaxed and ${\varvec{\Upsilon }}$ is estimated from the sample fourth-order moments, the estimator (12) is not necessarily equal to ${\hat{\varphi }}_{j}^{2}\left( {\varvec{S}}_{vz,j}^{T}{\varvec{S}}_{vv,j}^{-1}{\varvec{S}}_{vz,j}\right) ^{-1}$. For Proposition 1 to hold, we do not even need the MIIVs to be valid. Regardless of the validity of MIIVs, we always have ${\varvec{S}}_{vz,j}^{T}{\varvec{S}}_{vv,j}^{-1}\left( {\varvec{S}}_{vy,j}-{\varvec{S}}_{vz,j}\hat{{\varvec{\theta }}}_{1}^{(j)}\right) = {\varvec{0}}$, which makes the estimator (12) the same as ${\hat{\varphi }}_{j}^{2}\left( {\varvec{S}}_{vz,j}^{T}{\varvec{S}}_{vv,j}^{-1}{\varvec{S}}_{vz,j}\right) ^{-1}$. As revealed in the following proposition, if we relax the assumption that ${\varvec{\Upsilon }}$ is estimated by $2{\varvec{D}}^{+}\left( {\varvec{S}}\otimes {\varvec{S}}\right) {\varvec{D}}^{+T}$ but assume that all MIIVs are valid, the finite sample equivalence still holds.

#### Proposition 2

Suppose that all observed variables are continuous with finite fourth-order moments, Eq. (6) is correctly specified, and all MIIVs are valid. Then, ${\varvec{\Omega }}_j = \varphi _{j}^{2}{\varvec{\Sigma }}_{vv,j}$.

Proposition 2 implies that we can estimate ${\varvec{\Omega }}_j$ by ${\hat{\varphi }}_{j}^{2}{\varvec{S}}_{vv,j}$ to attain the finite sample equivalence of (12) and ${\hat{\varphi }}_{j}^{2}\left( {\varvec{S}}_{vz,j}^{T}{\varvec{S}}_{vv,j}^{-1}{\varvec{S}}_{vz,j}\right) ^{-1}$. It is also worth mentioning that Proposition 2 does not require the observed continuous variables to be normally distributed.

### Point Estimator of $\theta _{2}$

To estimate ${\varvec{\theta }}_{2}$ given $\hat{{\varvec{\theta }}}_{1}$, we minimize the least-squares fit function $T\left( \hat{{\varvec{\theta }}}_{1},{\varvec{\theta }}_{2}\right)$, where $T\left( {\varvec{\theta }}\right)$ is given by (4). The weight matrices commonly used for systemwide estimators can naturally be used here. Bollen and Maydeu-Olivares (2007) and Fisher and Bollen (2020) used ULS. It is worth mentioning that the estimator of ${\varvec{\theta }}_{2}$ is not robust against model misspecification, since we use the systemwide estimation here [see Bollen and Maydeu-Olivares (2007) page 321 and Nestler (2015b)]. It is also worth mentioning that non-convergence is not an issue when estimating ${\varvec{\theta }}_{1}$, since it is based on the closed-form estimator (7). However, when $T\left( \hat{{\varvec{\theta }}}_{1},{\varvec{\theta }}_{2}\right)$ is minimized to estimate ${\varvec{\theta }}_{2}$, non-convergence becomes a potential issue for the MIIV approach as well. However, we can always estimate $\hat{{\varvec{\theta }}}_{1}$ even if non-convergence prevents us from estimating ${\varvec{\theta }}_{2}$

### Standard Error of $\hat{\mathbf {\theta }}_{2}$

Bollen and Maydeu-Olivares (2007) derived the asymptotic distribution of the PIV estimator $\hat{{\varvec{\theta }}}_{2}$ under the ULS fit function. Their equation (31) was taken by Fisher and Bollen (2020) in equation (33). Under certain conditions, it is shown in the appendix that

14 $$\begin{aligned} \sqrt{n}\left( \hat{{\varvec{\theta }}}_{2}-{\varvec{\theta }}_{2}\right)= & {} {\varvec{C}}\left( {\varvec{\theta }},{\varvec{\sigma }}\right) \sqrt{n}\left( {\varvec{s}}-{\varvec{\sigma }}\right) +o_{\text {P}}\left( 1\right) , \end{aligned}$$

where ${\varvec{C}}\left( {\varvec{\theta }},{\varvec{\sigma }}\right) ={\varvec{H}}\left( {\varvec{\theta }}\right) \left[ {\varvec{I}}-{\varvec{J}}_{1}\left( {\varvec{\theta }}\right) {\varvec{K}}\left( {\varvec{\sigma }}\right) \right]$ with ${\varvec{H}}\left( {\varvec{\theta }}\right) =\left[ {\varvec{J}}_{2}^{T}\left( {\varvec{\theta }}\right) {\varvec{W}}{\varvec{J}}_{2}\left( {\varvec{\theta }}\right) \right] ^{-1}{\varvec{J}}_{2}^{T}\left( {\varvec{\theta }}\right) {\varvec{W}}$, ${\varvec{J}}_{1}\left( {\varvec{\theta }}\right) =\partial {\varvec{\sigma }}\left( {\varvec{\theta }}\right) /\partial {\varvec{\theta }}_{1}^{T}$ and ${\varvec{J}}_{2}\left( {\varvec{\theta }}\right) =\partial {\varvec{\sigma }}\left( {\varvec{\theta }}\right) /\partial {\varvec{\theta }}_{2}^{T}$. Expansion of (14) implies

15 $$\begin{aligned} \sqrt{n}\left( \hat{{\varvec{\theta }}}_{2}-{\varvec{\theta }}_{2}\right)&\overset{d}{\rightarrow }&N\left( {\varvec{0}},\;{\varvec{C}}\left( {\varvec{\theta }},{\varvec{\sigma }}\right) {\varvec{\Upsilon }}{\varvec{C}}^{T}\left( {\varvec{\theta }},{\varvec{\sigma }}\right) \right) . \end{aligned}$$

We estimate the standard errors from distribution (15) by evaluating ${\varvec{\theta }}$, ${\varvec{\sigma }}$, and ${\varvec{\Upsilon }}$ at $\hat{{\varvec{\theta }}}$, ${\varvec{s}}$, and $\hat{{\varvec{\Upsilon }}}$, respectively. Our ${\varvec{H}}$ matrix is more general than the ${\varvec{H}}$ matrix in equation (33) of Fisher and Bollen (2020) because we allow the use of a more general weight matrix ${\varvec{W}}$ to develop the standard errors whereas they assume ${\varvec{W=I}}$ as is true for ULS.^1^

## MIIV Tests

### Goodness-of-fit Tests for Model

Bollen and Maydeu-Olivares (2007) propose goodness-of-fit tests for the model as a whole for PIV. We develop similar model test statistics for the MIIV approach here. We show in the appendix that, if the model is correctly specified, $nT\left( {\varvec{\theta }}\right)$ evaluated at the MIIV estimates converges in distribution to a weighted sum of independent Chi-square random variables with 1 degree of freedom, where the weights are the eigenvalues of

16 $$\begin{aligned} {\varvec{M}}= & {} {\varvec{\Upsilon }}^{1/2} \left[ {\varvec{I}}-{\varvec{J}}\left( {\varvec{\theta }}\right) \begin{pmatrix}{\varvec{K}}\left( {\varvec{\sigma }}\right) \\ {\varvec{C}}\left( {\varvec{\theta }},{\varvec{\sigma }}\right) \end{pmatrix}\right] ^{T}{\varvec{W}}\left[ {\varvec{I}}-{\varvec{J}}\left( {\varvec{\theta }}\right) \begin{pmatrix}{\varvec{K}}\left( {\varvec{\sigma }}\right) \\ {\varvec{C}}\left( {\varvec{\theta }},{\varvec{\sigma }}\right) \end{pmatrix}\right] {\varvec{\Upsilon }}^{1/2}, \end{aligned}$$

with ${\varvec{J}}\left( {\varvec{\theta }}\right) =\partial {\varvec{\sigma }}\left( {\varvec{\theta }}\right) /\partial {\varvec{\theta }}^{T}$. Hence, we can apply the Satorra and Bentler (1994) adjustments to assess the overall goodness of fit of the model. In particular, the mean-scaled statistic and the mean-variance adjusted statistic are

$$\begin{aligned} T_{\mathrm{m}}=\frac{\mathrm{nr}}{\text {tr}\left\{ \hat{{\varvec{M}}}\right\} } T\left( \hat{{\varvec{\theta }}}\right)&\, \text { and } \,&T_{\mathrm{mv}}=\frac{n\text {tr}\left\{ \hat{{\varvec{M}}}\right\} }{\text {tr}\left\{ \hat{{\varvec{M}}}^{2}\right\} }T\left( \hat{{\varvec{\theta }}}\right) , \end{aligned}$$

respectively, where *r* is the difference between the number of nonredundant free entries in ${\varvec{\Sigma }}$ and the number of free parameters. The mean-scaled statistic is approximated by a Chi-square distribution with *r* degrees of freedom and the mean-variance adjusted statistic is approximated by a Chi-square distribution with $\left[ \text {tr}\left\{ \hat{{\varvec{M}}}\right\} \right] ^{2}/\text {tr}\left\{ \hat{{\varvec{M}}}^{2}\right\}$ degrees of freedom.

### Overidentification Test for Equations

We mentioned earlier that an advantage of the MIIV approach is that we have an overidentification tests for every overidentified equation in the system (5). This provides a test of the validity of the MIIVs and the validity of the model specification. The reader is directed to Bollen (2019) for the implications of such a test. In this section, we present the asymptotic Chi-square distributed overidentification test that is applicable to continuous, ordinal, and binary endogenous observed variables. The derivation by and large follows the derivation in Jin and Cao (2018).

If equation (6) is correctly specified and the MIIVs are valid, ${\varvec{g}}_j \left( {\varvec{\sigma }}\right) ={\varvec{0}}$. Then the delta method indicates that

17 $$\begin{aligned} \sqrt{n}\left( {\varvec{S}}_{vy,j}-{\varvec{S}}_{vz,j}{\varvec{\theta }}_{1}^{(j)}\right) =\sqrt{n}\left( {\varvec{g}}_j\left( {\varvec{s}}\right) -{\varvec{g}}_j\left( {\varvec{\sigma }}\right) \right)&\overset{d}{\rightarrow }N\left( {\varvec{0}},{\varvec{\Omega }}\right) . \end{aligned}$$

It is easy to show that

$$\begin{aligned} {\varvec{Q}}_j = {\varvec{I}}-{\varvec{\Omega }}_j^{-1/2}{\varvec{\Sigma }}_{vz,j}\left( {\varvec{\Sigma }}_{vz,j}^{T}{\varvec{\Sigma }}_{vv,j}^{-1}{\varvec{\Sigma }}_{vz,j}\right) ^{-1}{\varvec{\Sigma }}_{vz,j}^{T}{\varvec{\Sigma }}_{vv,j}^{-1}{\varvec{\Omega }}_j^{1/2}&. \end{aligned}$$

is an idempotent matrix, but not necessarily symmetric. Let $\hat{{\varvec{Q}}}_j$ be an estimator of ${\varvec{Q}}_j$ by replacing ${\varvec{\Sigma }}$ with ${\varvec{S}}$ and ${\varvec{\theta }}_1^{(j)}$ with $\hat{{\varvec{\theta }}}_1^{(j)}$. Together with Eq. (7), it can be shown that

18 $$\begin{aligned} \sqrt{n}\hat{{\varvec{\Omega }}}_j^{-1/2}\left( {\varvec{S}}_{vy,j}-{\varvec{S}}_{vz,j}\hat{{\varvec{\theta }}}_{1}^{(j)}\right) =&\sqrt{n}\hat{{\varvec{Q}}}_j \hat{{\varvec{\Omega }}}_j^{-1/2}{\varvec{S}}_{vy,j}=\sqrt{n}\hat{{\varvec{Q}}}_j \hat{{\varvec{\Omega }}}_j^{-1/2}\left( {\varvec{S}}_{vy,j}-{\varvec{S}}_{vz,j}{\varvec{\theta }}_1^{(j)}\right) , \end{aligned}$$

where the second equality holds since $\sqrt{n}\hat{{\varvec{Q}}}_j\hat{{\varvec{\Omega }}}_j^{-1/2}{\varvec{S}}_{vz} = {\varvec{0}}$. However, the asymptotic variance of (18) is ${\varvec{Q}}_j{\varvec{Q}}_j^{T}$, which is not of full rank. Let ${\varvec{G}}_j$ and $\hat{{\varvec{G}}}_j$ be the Moore–Penrose inverses of ${\varvec{Q}}_j{\varvec{Q}}_j^{T}$ and $\hat{{\varvec{Q}}}_j\hat{{\varvec{Q}}}_j^{T}$, respectively. The following theorem shows that

19 $$\begin{aligned} F = n\left( {\varvec{S}}_{vy,j}-{\varvec{S}}_{vz,j}\hat{{\varvec{\theta }}}_1^{(j)}\right) ^{T}\hat{{\varvec{\Omega }}}_j^{-1/2}\hat{{\varvec{G}}}_j\hat{{\varvec{\Omega }}}_j^{-1/2}\left( {\varvec{S}}_{vy,j}-{\varvec{S}}_{vz,j}\hat{{\varvec{\theta }}}_1^{(j)}\right) \end{aligned}$$

is asymptotically Chi-square distributed.

#### Theorem 1

As $n\rightarrow \infty$, *F* converges in distribution to a Chi-square distribution with $L_{j}-K_{j}$ degrees of freedom, provided that Eq. (6) is correctly specified and all MIIVs are valid, where $L_{j}$ is the number of MIIVs, $K_{j}$ is the number of explanatory variables in $\tilde{{\varvec{z}}}_{j}$, and $L_{j}-K_{j}>0$.

The expression of *F* is the same as the equation (9) in Jin and Cao (2018) for observed ordinal variables. We have shown in Theorem 1 that it is also valid for a mixture of different types of variables. We can interpret the test statistic *F* as the generalized Wald statistic of Andrews (1987).

### Connection with Sargan’s Chi-Square

The test statistic (19) is asymptotically Chi-square in general, including the case where all observed variables are continuous. The Sargan’s Chi-square statistic

$$\begin{aligned} F_{\mathrm{Sargan}}&=\frac{{\varvec{e}}_{j}^{T}{\varvec{V}}_{j}\left( {\varvec{V}}_{j}^{T}{\varvec{V}}_{j}\right) ^{-1}{\varvec{V}}_{j}^{T}{\varvec{e}}_{j}}{{\varvec{e}}_{j}^{T}{\varvec{e}}_{j}/n}\,, \end{aligned}$$

applies when all observed variables are continuous, where ${\varvec{e}}_{j}={\varvec{y}}_{j}-{\varvec{Z}}_{j}\hat{{\varvec{\theta }}}_1^{(j)}$ is the residual vector from Eq. (6). Equivalently,

20 $$\begin{aligned} F_{\mathrm{Sargan}}&=n\left( {\varvec{S}}_{vy,j}-{\varvec{S}}_{vz,j}\hat{{\varvec{\theta }}}_1^{(j)}\right) ^{T}\frac{{\varvec{S}}_{vv,j}^{-1}}{{\hat{\varphi }}_{j}^{2}}\left( {\varvec{S}}_{vy,j}-{\varvec{S}}_{vz,j}\hat{{\varvec{\theta }}}_1^{(j)}\right) . \end{aligned}$$

The asymptotic distribution of $F_{Sargan}$ is derived by Sargan (1958). It is not surprising that *F* and $F_{Sargan}$ are asymptotically equivalent, if all observed variables are continuous. The asymptotic distributions of *F* and $F_{Sargan}$ essentially depend on the distribution of $\sqrt{n}\left( {\varvec{S}}_{vy,j}-{\varvec{S}}_{vz,j}{\varvec{\theta }}_1^{(j)}\right)$. If all observed variables are continuous and all MIIVs are valid,

$$\begin{aligned} \sqrt{n}\left( {\varvec{S}}_{vy,j}-{\varvec{S}}_{vz,j}{\varvec{\theta }}_1^{(j)}\right) =&\sqrt{n}\left( \frac{1}{n}{\varvec{V}}_{j}^{T}{\varvec{y}}_{j}-\frac{1}{n}{\varvec{V}}_{j}^{T}{\varvec{Z}}_{j}{\varvec{\theta }}_1^{(j)}\right) \overset{d}{\rightarrow }N\left( {\varvec{0}},\varphi _{j}^{2}{\varvec{\Sigma }}_{vv,j}\right) . \end{aligned}$$

Otherwise, the distribution (17) applies.

Despite of the asymptotic equivalence, *F* and $F_{Sargan}$ are not necessarily the same due to the finite sample estimator of ${\varvec{\Omega }}_j$. A properly chosen estimator of ${\varvec{\Omega }}_j^{-1/2}{\varvec{G}}_j{\varvec{\Omega }}_j^{-1/2}$ is needed to yield the established test statistics in the literature. By Proposition 2, if ${\varvec{\Omega }}_j$ is estimated by ${\hat{\varphi }}_{j}^{2}{\varvec{S}}_{vv,j}$, then $F = F_{\mathrm{Sargan}}$ at any sample size. Alternatively, ${\varvec{\Omega }}_j$ can be estimated by

$$\begin{aligned} \tilde{{\varvec{\Omega }}}_j = \hat{{\varvec{\Omega }}}_j - \left( {\varvec{S}}_{vy,j}-{\varvec{S}}_{vz,j}\hat{{\varvec{\theta }}}_1^{(j)}\right) \left( {\varvec{S}}_{vy,j}-{\varvec{S}}_{vz,j}\hat{{\varvec{\theta }}}_1^{(j)}\right) ^{T}. \end{aligned}$$

Consequently,

21 $$\begin{aligned} {\tilde{F}} = n\left( {\varvec{S}}_{vy,j}-{\varvec{S}}_{vz,j}\hat{{\varvec{\theta }}}_1^{(j)}\right) ^{T}\tilde{{\varvec{\Omega }}}_j^{-1/2}\tilde{{\varvec{G}}}_j \tilde{{\varvec{\Omega }}}_j^{-1/2}\left( {\varvec{S}}_{vy,j}-{\varvec{S}}_{vz,j}\hat{{\varvec{\theta }}}_1^{(j)}\right) \end{aligned}$$

is asymptotically the same as *F*, where $\tilde{{\varvec{G}}}_j$ is the Moore–Penrose inverse of $\tilde{{\varvec{Q}}}_j \tilde{{\varvec{Q}}}_j^{T}$ with

22 $$\begin{aligned} \tilde{{\varvec{Q}}}_j=&{\varvec{I}}-\tilde{{\varvec{\Omega }}}_j^{-1/2}{\varvec{S}}_{vz,j}\left( {\varvec{S}}_{vz,j}^{T}{\varvec{S}}_{vv,j}^{-1}{\varvec{S}}_{vz,j}\right) ^{-1}{\varvec{S}}_{vz,j}^{T}{\varvec{S}}_{vv,j}^{-1}\tilde{{\varvec{\Omega }}}_j^{1/2}. \end{aligned}$$

If all variables are normal and $\hat{{\varvec{\Upsilon }}} = 2{\varvec{D}}^{+}\left( {\varvec{S}}\otimes {\varvec{S}}\right) {\varvec{D}}^{+T}$, then $\tilde{{\varvec{\Omega }}}_j$ reduces to ${\hat{\varphi }}_{j}^{2}{\varvec{S}}_{vv,j}$ (Proposition 1) and ${\tilde{F}}$ is exactly the same as $F_{Sargan}$ at any sample size.

### Satorra-Bentler Adjusted Overidentification Tests of Equations

Besides the Chi-square distributed test statistic, Jin and Cao (2018) proposed alternative test statistics in the spirit of Satorra and Bentler (1994) and showed that the Satorra–Bentler-based statistics tend to have better small sample properties than the Chi-square distributed test statistic. In this section, the test statistic in Jin and Cao (2018) is extended to the mixture of different types of variables as well.

We can express the Sargan’s Chi-square statistic $F_{\mathrm{Sargan}}$ as

23 $$\begin{aligned} F_{\mathrm{Sargan}}= & {} \frac{n\left( {\varvec{S}}_{vy,j}-{\varvec{S}}_{vz,j}\hat{{\varvec{\theta }}}_1^{(j)}\right) ^{T}{\varvec{S}}_{vv,j}^{-1}\left( {\varvec{S}}_{vy,j}-{\varvec{S}}_{vz,j}\hat{{\varvec{\theta }}}_1^{(j)}\right) }{{\varvec{S}}_{yy,j}-\hat{{\varvec{\theta }}}_1^{(j)T}{\varvec{S}}_{zy,j}-{\varvec{S}}_{zy,j}^{T}\hat{{\varvec{\theta }}}_1^{(j)}+\hat{{\varvec{\theta }}}_1^{(j)T}{\varvec{S}}_{zz,j}\hat{{\varvec{\theta }}}_1^{(j)}} . \end{aligned}$$

Jin and Cao (2018) termed (23) as “Naive Sargan’s Chi-square statistic” and showed that its asymptotic distribution is a weighted sum of independent Chi-square random variables with 1 degrees of freedom, if all observed variables are ordinal. The next theorem generalizes their results.

#### Theorem 2

The asymptotic distribution of $F_{\mathrm{Sargan}}$ is a weighted sum of independent Chi-square random variables with 1 degrees of freedom, if all MIIVs are valid. The weights are the eigenvalues of ${\varvec{\Pi }}$, where

$$\begin{aligned} {\varvec{\Pi }}_j= & {} \varphi _{j}^{-2}{\varvec{\Omega }}_j^{1/2}\left[ {\varvec{\Sigma }}_{vv,j}^{-1}-{\varvec{\Sigma }}_{vv,j}^{-1}{\varvec{\Sigma }}_{vz,j}\left( {\varvec{\Sigma }}_{vz,j}^{T}{\varvec{\Sigma }}_{vv,j}^{-1}{\varvec{\Sigma }}_{vz,j}\right) ^{-1}{\varvec{\Sigma }}_{vz,j}^{T}{\varvec{\Sigma }}_{vv,j}^{-1}\right] {\varvec{\Omega }}_j^{1/2}. \end{aligned}$$

Theorem 2 implies that we can apply the Satorra–Bentler-type adjustments to $F_{\mathrm{Sargan}}$. In particular, the mean-scaled statistic and and the mean-variance adjusted statistic are

$$\begin{aligned} F_{\mathrm{m}} = \frac{L_{j}-K_{j}}{\text {tr}\left\{ \hat{{\varvec{\Pi }}}_j \right\} }F_{\mathrm{Sargan}}&\text { and }&F_{mv} = \frac{\text {tr}\left\{ \hat{{\varvec{\Pi }}}_j \right\} }{\text {tr}\left\{ \hat{{\varvec{\Pi }}}_j ^{2}\right\} }F_{\mathrm{Sargan}}, \end{aligned}$$

respectively, where $\hat{{\varvec{\Pi }}}_j$ is the estimator of ${\varvec{\Pi }}_j$, replacing ${\varvec{\Sigma }}$ by ${\varvec{S}}$ and ${\varvec{\theta }}_1^{(j)}$ by $\hat{{\varvec{\theta }}}_1^{(j)}$. We can approximate the mean-scaled statistic by a Chi-square distribution with $L_{j}-K_{j}$ degrees of freedom and approximate the mean-variance adjusted statistic by a Chi-square distribution with $\left[ \text {tr}\left\{ \hat{{\varvec{\Pi }}}_j\right\} \right] ^{2}/\text {tr}\left\{ \hat{{\varvec{\Pi }}}_j^{2}\right\}$ degrees of freedom.

If the observed variables are ordinal, the mean-scaled and mean-variance adjusted statistics derived by Jin and Cao (2018) are

24 $$\begin{aligned}&\frac{L_{j}-K_{j}}{\text {tr}\left( \hat{{\varvec{\Delta }}}_j\right) }F_{\mathrm{Sargan}}\quad \text { and }\quad \frac{\text {tr}\left\{ \hat{{\varvec{\Delta }}}_j\right\} }{\text {tr}\left\{ \hat{{\varvec{\Delta }}}_j^{2}\right\} }F_{\mathrm{Sargan}}, \end{aligned}$$

respectively, where $\hat{{\varvec{\Delta }}}_j$ is an estimator of

$$\begin{aligned} {\varvec{\Delta }}_j&=\frac{1}{1-{\varvec{\Sigma }}_{yz,j}{\varvec{\theta }}_1^{(j)}-{\varvec{\theta }}_1^{(j)T}{\varvec{\Sigma }}_{yz,j}^{T}+{\varvec{\theta }}_1^{(j)T}{\varvec{\Sigma }}_{zz,j}{\varvec{\theta }}_1^{(j)}}{\varvec{\Sigma }}_{vv,j}^{-1/2}\left( \frac{\partial {\varvec{h}}_j\left( {\varvec{{\varvec{\sigma }}}}\right) }{\partial {\varvec{{\varvec{\sigma }}}}^{T}}\right) {\varvec{\Upsilon }}\left( \frac{\partial {\varvec{h}}_j\left( {\varvec{{\varvec{\sigma }}}}\right) }{\partial {\varvec{{\varvec{\sigma }}}}^{T}}\right) ^{T}{\varvec{\Sigma }}_{vv,j}^{-1/2} \end{aligned}$$

with ${\varvec{h}}_j\left( {\varvec{\sigma }}\right) ={\varvec{\Sigma }}_{vy,j}-{\varvec{\Sigma }}_{vz,j}{\varvec{\gamma }}_{j}\left( {\varvec{\sigma }}\right)$. Despite that ${\varvec{\Pi }}_j$ is not necessarily the same as ${\varvec{\Delta }}_j$, the following corollary shows their connections.

#### Corollary 1

$tr\left\{ {\varvec{\Delta }}_j \right\} =tr\left\{ {\varvec{\Pi }}_j \right\}$ and $tr\left\{ {\varvec{\Delta }}_j^{2}\right\} =tr\left\{ {\varvec{\Pi }}_j^{2}\right\}$ provided that observed variables are ordinal and all MIIVs are valid. However, their finite sample estimators differ by a function of ${\varvec{h}}_j\left( {\varvec{s}}\right)$.

Corollary 1 indicates that $F_{\mathrm{m}}$ and $F_{\mathrm{mv}}$ are asymptotically the same as the statistics developed by Jin and Cao (2018), if only ordinal variables are observed and all MIIVs are valid. If all observed variables are continuous and all MIIVs are valid, ${\varvec{\Pi }}_j={\varvec{Q}}_j$, and $F_{\mathrm{m}}$, $F_{\mathrm{mv}}$, and $F_{\mathrm{Sargan}}$ are asymptotically the same. The implication is that $F_{\mathrm{m}}$ and $F_{\mathrm{mv}}$ can always be computed as test statistics and be approximated by their corresponding Chi-square distributions. Similarly to the Chi-square distributed test statistic, the consistent estimator $\tilde{{\varvec{\Omega }}}_j$ needs to be used to ensure small sample equivalence of $F_{\mathrm{m}}$, $F_{\mathrm{mv}}$, and $F_{\mathrm{Sargan}}$ under the assumptions in Proposition 1, whereas $\hat{{\varvec{\Omega }}}_j$ only ensures asymptotic equivalence. Hereafter, the mean-scaled statistic and mean-variance adjusted statistic using $\tilde{{\varvec{\Omega }}}_j$ are denoted by ${\tilde{F}}_{\mathrm{m}}$ and ${\tilde{F}}_{\mathrm{mv}}$, respectively.

## Monte Carlo Simulation

In this section a simulation study is conducted to investigate the finite sample properties of the proposed MIIV approach. The simulation is performed in R (R Core Team, 2020) and is built on the packages lavaan (Rosseel, 2012) and MIIVsem (Fisher et al., 2017).^2^ Due to space limitation, we present only limited results in the subsequent section.

### Simulation Design

**Fig. 1:**
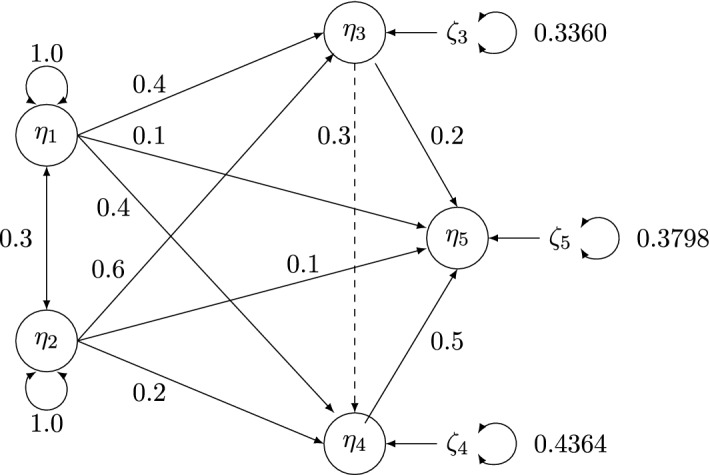
Path diagram of the latent regression part of the SEM model in the simulation study. The dashed line is present in the true model but is omitted in the misspecified model. The population values are the standardized coefficients.

To explore some of our proposed statistics, we use the SEM model in Li (2016) with two exogenous and three endogenous latent variables. The latent regression of the true data generating process with standardized coefficients is shown in Fig. 1. Every latent variable is measured by three indicators. The indicators of $\eta _1$, $\eta _3$, and $\eta _5$ are continuous, whereas the indicators of $\eta _2$ and $\eta _4$ are ordinal with five categories. The response probabilities are 0.04, 0.05, 0.21, 0.46, and 0.24, which is the slightly asymmetric condition in Li (2016). Each ordinal variable has an underlying continuous variable, and we set the error variances of the latter so that their total variances equal one. For simplicity, we also let the variances of continuous indicators equal one, but the error variances of continuous indicators are freely estimated. The factor loadings are set to 0.8, 0.65, and 0.5 with corresponding $R^2$ of indicators being 0.64, 0.42, and 0.25.

Given the scaling indicator, MIIVs are automatically generated from the hypothesized model using the package MIIVsem. Previous studies (e.g., Bollen et al., 2007; Jin and Cao, 2018) suggested that when the sample size is small, using fewer IVs results in less bias than using all IVs. Hence, we consider three estimators in the simulation study, i.e., MIIV with one more IV than the number of endogenous variables (denoted by MIIV1), MIIV with all possible IVs (denoted by MIIVall), and DWLS. We use the same set of MIIVs for estimation and in the model goodness-of-fit tests and the equation overidentification tests.

**Fig. 2:**
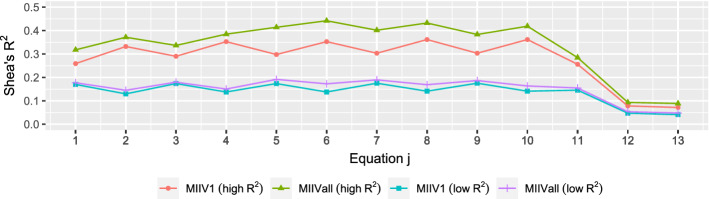
Shea (1997)’s $R^2$ for each equation in the population model.

Since the MIIV approach uses the scaling indicators to set the scale of ${\varvec{\eta }}$, we explore two choices of the scaling indicator, i.e., the indicators with standardized factor loadings 0.8 and the indicators with standardized factor loadings 0.5. The former sets the scale of ${\varvec{\eta }}$ using the indicator with the highest $R^2$, whereas the latter uses the indicator with the lowest $R^2$. We also use the same MIIV scaling indicators for DWLS, so that we can compare the effects of scaling on different estimators. Fisher and Bollen (2020) have shown that the scaling indicator with a higher Shea (1997)’s $R^2$ contributes to lower biases in the MIIV approach. The $R^2$ of the scaling indicator measures the reliability of that indicator, whereas the Shea (1997)’s $R^2$ is a diagnostic for “weak" instrumental variables. Choosing the scaling indicator as the one with the lowest reliability or lowest Shea (1997)’s $R^2$ weakens the instrument compared to choosing the scaling indicator with the higher values. MIIVall uses all MIIVs in estimation. MIIV1 uses one more than the minimum number of MIIVs and these are chosen to maximize the Shea (1997)’s $R^2$. Figure 2 illustrates the values of Shea (1997)’s $R^2$ for the data generating process. There are ten equations for factor loadings and three equations for latent regression coefficients in our model. Figure 2 shows that the maximum Shea (1997)’s $R^2$ never exceeds 0.5 and the highest values occur when the scaling indicator has the highest $R^2$ value. The Shea (1997)’s $R^2$ is considerably lower for the scaling indicators with the lowest $R^2$s. Indeed for two of these equations, the Shea (1997)’s $R^2$ value is lower than 0.1. Although the high $R^2$ scaling indicator condition generally exceeds the low $R^2$ scaling indicator condition, the last two equations for the latent variable model have values of 0.1 or less. Hence, the simulation allows us to examine how very weak instrumental variables affect the estimates.

We fit both correctly specified and misspecified models. The hypothesized model is correctly specified, if it includes all paths in Figure 1. The misspecified model omits the dashed path $b_{43}$. For each model, six sample sizes are considered, namely, $n=200$, 400, 800, 1200, 2000, and 3200. The number of replication is 10, 000, for each combination of the hypothesized model, sample size and choice of scaling indicator.

### Outcome Measures

If only the coefficients and factor loadings of ${\varvec{\theta }}_1$ are of interest, MIIV always converges. Non-convergence is only a potential problem for MIIV when ${\varvec{\theta }}_2$ is estimated, but it is always a potential problem for DWLS. In the current study, ${\varvec{\theta }}_2$ is estimated using the DWLS fit function, given the MIIV estimator of ${\varvec{\theta }}_1$. Even for the converged estimates, the covariance matrices of error terms and factors are possibly non-positive definite. Both non-convergence and non-positive definite solutions are regarded as improper and are discarded in the current study.

To investigate the accuracy of the MIIV estimator, we compute the relative bias $100 \times \text {median of } \left\{ \theta ^{-1} \left( {\hat{\theta }}_{r}- \theta \right) , r = 1, ..., R \right\}$, where ${\hat{\theta }}_{r}$ is the estimate of $\theta$ in the *r*th replication and *R* is the number of proper replications. We use the median instead of the mean to prevent outliers from affecting the central tendency estimate in the smaller sample sizes. We focus on relative bias greater than 5 percent bias. The relative bias is also used to investigate the accuracy of the standard error estimates. However, we do not know the true value of the standard error. Hence, we use the sample standard deviation of the estimates as the pseudo-true value. Due to the presence of outliers, we use the interquartile range to trim the estimates, where the interquartile range is calculated by the third empirical quartile minus the first empirical quartile. Any estimate of a parameter is trimmed if the estimate is less than the first empirical quartile minus 3 times the interquartile range or larger than the third empirical quartile plus the 3 times the interquartile range. Since the converged replications with negative definite covariance matrices also reflect sampling fluctuation, the standard deviation of the converged estimates after trimming is treated as robust estimate of the standard error.

Concerning the goodness-of-fit test for the model as a whole, both the mean scaled test $T_\mathrm{m}$ and the mean-variance adjusted test $T_{\mathrm{mv}}$ are investigated. For DWLS, only the mean-scaled test is used. The goodness-of-fit tests of models are only computed for proper solutions. Regarding the overidentification tests for equations, the Chi-square tests ((19) and (21)), the mean scaled tests ($F_\mathrm{m}$ and ${\tilde{F}}_\mathrm{m}$), and the mean-variance adjusted tests ($F_{\mathrm{mv}}$ and ${\tilde{F}}_{\mathrm{mv}}$) are investigated. We will only focus on the overidentification equation tests for the misspecified model, since it contains both correctly and incorrectly specified equations when estimating ${\varvec{\theta }}_1$. Because the overidentification equation tests does not require estimation of ${\varvec{\theta }}_2$, we compute them for all converged solutions, including the solutions with negative covariance matrices. We use the empirical percentage of test statistics that exceed the $\chi ^{2}$ critical value at the significance level 0.05 for all test statistics.

### Percentage of Proper Solutions

Table 1 shows that MIIV and DWLS generally have similar percentages of proper solutions when the $R^2$ of the scaling indicator is high. If the $R^2$ is low and *n* is low, MIIV can yield more improper solutions than DWLS. Recall that MIIV always converges if only ${\varvec{\theta }}_1$ is estimated. Hence, the improper solutions for MIIV originates from estimating ${\varvec{\theta }}_2$.

**Table 1:** Percentage of converged solutions with positive definite covariance matrices.

Model	$R^{2}$	Method	Sample size				
			200	400	800	1200	2000
Correctly specified	High	DWLS	99.15	100.00	100.00	100.00	100.00
		MIIV1	96.49	99.84	100.00	100.00	100.00
		MIIVall	98.56	99.97	100.00	100.00	100.00
	Low	DWLS	99.20	99.99	100.00	100.00	100.00
		MIIV1	87.70	98.22	99.96	99.97	100.00
		MIIVall	89.45	98.29	99.96	100.00	100.00
Misspecified	High	DWLS	98.46	99.97	100.00	100.00	100.00
		MIIV1	96.23	99.84	100.00	100.00	100.00
		MIIVall	98.74	99.99	100.00	100.00	100.00
	Low	DWLS	98.44	99.99	100.00	100.00	100.00
		MIIV1	87.55	97.77	99.86	100.00	100.00
		MIIVall	91.00	98.47	99.94	100.00	100.00

### Correctly Specified Model

**Fig. 3:**
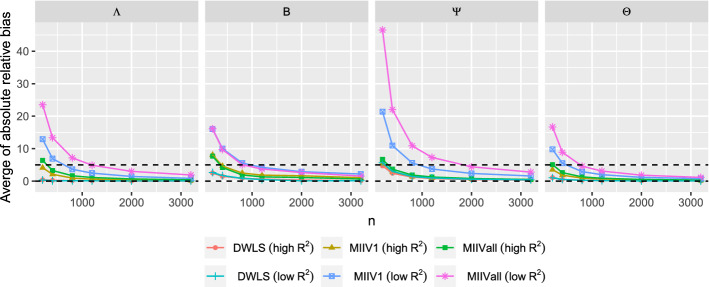
Averaged absolute value of the relative bias of the parameter estimators when the model is correctly specified. Dashed lines at 0 and 5 percent relative bias.

When the model is correctly specified, all estimators are consistent estimators. We report the average of the absolute value of the relative bias for each of the four parameter sets (${\varvec{\Lambda }}$, ${\varvec{B}}$, ${\varvec{\Psi }}$, and ${\varvec{\Theta }}$). Figure 3 shows that the biases of all estimators converges toward zero as sample size grows. Under the high $R^2$ condition, the average absolute value of the relative bias of the parameter estimates is low for DWLS, MIIVall and MIIV1. In contrast, when both the $R^2$ and sample size are low, DWLS maintains low bias, while MIIVall and MIIV1 exhibit more bias that diminishes as the sample size increases. It is also clear that MIIV1 tends to be less biased than MIIVall, especially when the scaling indicator has a low $R^2$. When the $R^2$ is low, MIIVall has the highest average absolute value of the relative bias for the latent variable equation error variances (${\varvec{\Psi }}$).

**Fig. 4:**
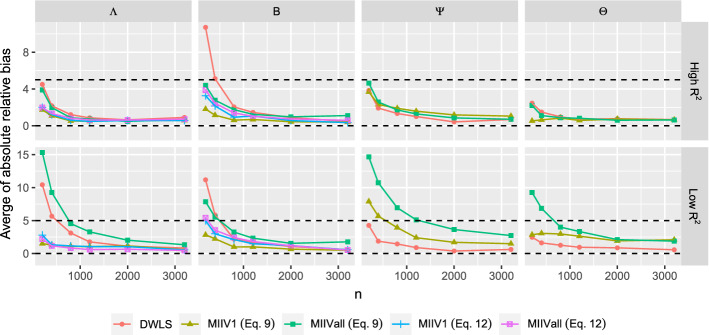
Averaged absolute value of the relative bias of the standard error estimators when the model is correctly specified. Note: The MIIV standard errors of $\hat{{\varvec{\Lambda }}}$ and $\hat{{\varvec{B}}}$ are computed from Eq. 9 or 12. The MIIV standard errors of $\hat{{\varvec{\Psi }}}$ and $\hat{{\varvec{\Theta }}}$ are computed from Eq. 15, hence MIIV Eq. 9 is the same as MIIV Eq.  12.

Figure 4 reveals the averaged absolute value of relative bias of the standard error estimates for each parameter set. We found some extreme outliers at the smaller sample sizes, so we trimmed values that were greater than three times the interquartile range above the third quartile or below the first quartile. This resulted in no more than $1.3 \%$ of cases trimmed which occurred when $n=200$. The percentage trimmed rapidly decreased toward zero as *n* increased. For MIIV, both standard errors from (9) and (12) are expected to be accurate in the correctly specified model. If the $R^2$ of the scaling indicator is high, all standard errors are accurate. The only exception is that the relative bias of the standard errors is larger for the latent variable regression coefficients (${\varvec{B}}$) for DWLS at the smaller sample size. If the $R^2$ is low, the pattern is more complex. Under these conditions, the relative bias of the standard errors of the factor loadings and latent variable regression coefficients are lowest for MIIV1 (Eq. 9), MIIV1 (Eq. 12), and MIIVall (Eq. 12). The bias is greatest for MIIVall (equation 9) and DWLS, though these biases diminish as sample size increases. Continuing with the low $R^2$ results, the bias of the standard errors of the variance parameter estimates are highest for MIIVall followed by MIIV1 and DWLS. These too diminish as the sample size increases. When MIIV1 is used, the standard error estimator (12) tends to be similar to (9). However, the standard error estimator (9) tends to be more biased than (12) for MIIVall.

**Fig. 5:**
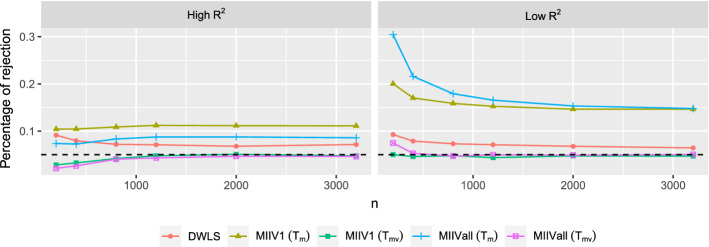
Percentages of rejection of the goodness-of-fit tests when the model is correctly specified. The significance level is 0.05 (dashed line).

Given that the model is correctly specified, we can explore the empirical size of the goodness-of-fit tests for models by computing the percentage of test statistics that exceed the critical value of a Chi-square with significance level 0.05. Figure 5 shows that using different number of IVs does not have strong effects on the empirical size. The mean-variance adjusted statistics for MIIV are the closest to the nominal level of all of the model test statistic (Jin et al., 2016). The size of the mean-scaled statistic for DWLS tends to be too high at about the same magnitude for the high and low $R^2$ scaling indicators. The mean-scaled statistics for MIIV are the least accurate, tending to be somewhat higher under the high $R^2$ condition and much higher under the low $R^2$ condition.

### Misspecified Model

In the misspecified model, $b_{43}$ is mistakenly fixed to 0, indicating that the latent regression with $\eta _4$ as the dependent variable ($j=12$) is misspecified. Bollen (2001) and Bollen et al. (2018a) give the conditions under which the MIIV-2SLS and PIV estimator of coefficients will be robust under structural misspecifications such as in this model. Using their results, we know that MIIV factor loading (${\varvec{\Lambda }}$) estimates are identical under the correct and incorrect specifications and hence will have identical properties. Similarly, the MIIV estimates of the coefficients of $b_{51}$, $b_{52}$, $b_{53}$ and $b _{54}$ from the $\eta _5$ equation are robust to the structural misspecification. In contrast, the MIIV estimates of $b_{41}$ and $b_{42}$ from the latent regression for $\eta _4$ are not robust due to the omission of $\eta _3$ from the $\eta _4$ equation. In addition, the MIIV estimates of $b_{31}$ and $b _{32}$ from the latent regression for $\eta _3$ (with $j=11$) are not robust. The reason is that in the misspecified model, it mistakenly appears that $y_{10}$, $y_{11}$, and $y_{12}$, the indicators of $\eta _4$, are suitable MIIVs because the path from $\zeta _3$ to these indicators is cutoff by setting $b_{43}$ to zero. Hence, we expect MIIVall to be consistent for ${\varvec{\Lambda }}$, $b_{51}$, $b_{52}$, $b_{43}$ and $b _{54}$, but inconsistent for $b_{31}$, $b _{32}$, $b_{41}$, and $b _{42}$. In contrast, MIIV1 can be consistent for $b_{31}$ and $b _{32}$ as long as $y_{10}$, $y_{11}$, and $y_{12}$ are excluded. These robustness conditions describe the status of the factor loadings and latent variable coefficients, but there are no analogous analytic conditions for the variance parameters.

**Fig. 6:**
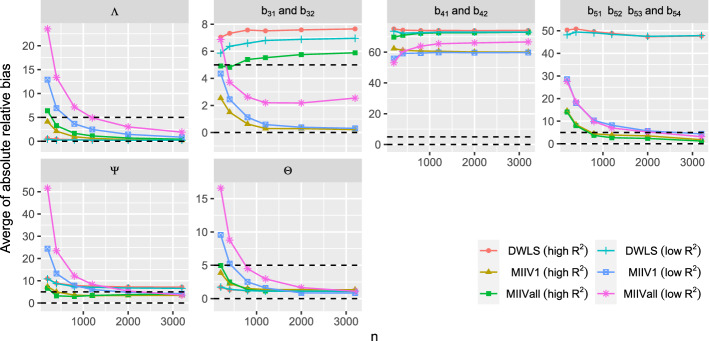
Averaged absolute value of the relative bias of the parameter estimators when the model is misspecified.

MIIV1 and MIIVall versions of MIIV are robust in the estimation of the factor loadings (${\varvec{\Lambda }}$). Therefore, the results of estimating the factor loadings for MIIV1 and MIIVall should be the same in the misspecified as they were in the correctly specified model, since the misspecification in the latent regression model does not affect the MIIVs for the measurement model. Figure 6 confirms this. Both MIIV1 and MIIVall yield consistent estimators of ${\varvec{\Lambda }}$ regardless of the $R^2$ of the scaling indicator. DWLS does not seem to spread the bias to the measurement model and in the smaller samples the bias is even lower than that of MIIV1 and MIIVall, though the differences are slight in the bigger samples. In contrast, DWLS spreads the bias over all regression coefficients from the entire latent regression part. The DWLS average of absolute bias ranges from a low of 6 to 8 percentage bias for $b_{31}$ and $b _{32}$ to 50 percent bias for $b_{51}$, $b_{52}$, $b_{53}$ and $b _{54}$ and 70 percent bias for $b_{41}$ and $b_{42}$. The MIIV1 and MIIVall estimators of $b_{51}$, $b_{52}$, $b_{53}$ and $b _{54}$ have large small sample biases, but decrease toward zero as *n* increases. The MIIV1 estimates of $b_{31}$ and $b _{32}$ have low biases, whereas the MIIVall estimator has a large bias if the scaling indicator has a large $R^2$. It is however interesting to see that the small sample bias of MIIVall is low if the scaling indicator has a low $R^2$. Hence, the MIIVall estimator of $b_{31}$ and $b _{32}$ can be inconsistent, but the asymptotic bias can still be low. When it comes to the effect of the scaling indicator, using a strong scaling indicator generally yields a lower bias than using a weak one for all parameters that can be consistently estimated.

**Fig. 7:**
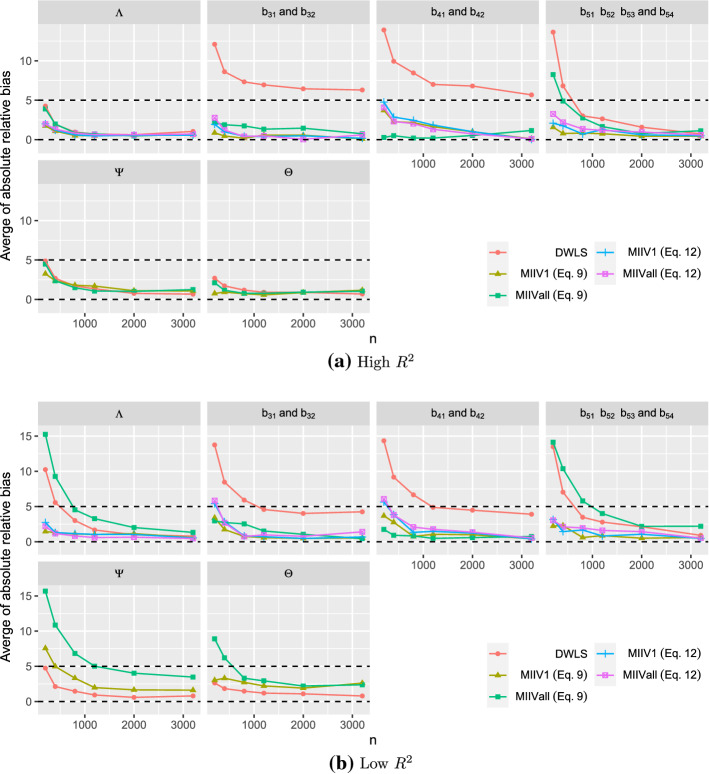
Averaged absolute value of the relative bias of the standard error estimators when the model is misspecified and the $R^2$ of the scaling indicator is high. Note: The MIIV standard errors of $\hat{{\varvec{\Lambda }}}$ and $\hat{{\varvec{B}}}$ are computed from equation (9) or (12). The MIIV standard errors of $\hat{{\varvec{\Psi }}}$ and $\hat{{\varvec{\Theta }}}$ are computed from Eq. 15, hence MIIVEq. 9 is the same as MIIV(Eq. 12).

If the model is misspecified, we still expect the standard errors from Eq. (9) to correctly quantify the variation of $\hat{{\varvec{\theta }}}_1$, even though ${\hat{b}}_{31}$, ${\hat{b}}_{32}$, ${\hat{b}}_{41}$, and ${\hat{b}}_{42}$ are not consistent estimators of the true population value. However, the standard errors from (12) can be biased for these same coefficients, since it needs the MIIVs to be valid. From Eq. 10, if ${\varvec{S}}_{vy,j} - {\varvec{S}}_{\mathrm{vz},j}{\varvec{\gamma }}_{j}\left( {\varvec{s}}\right)$ is far away from ${\varvec{0}}$, the bias is large. Otherwise the bias is small. It is seen from Figure 7 that all MIIV standard errors have low biases as *n* increases, regardless of the $R^2$. In contrast, the DWLS standard errors tend to be biased for the estimates of *B*. It is interesting to see that the standard error (12) also yields a low bias when the MIIVs are invalid. Nevertheless, we still prefer standard errors from Eq. 9, as it is theoretically valid. The bias of estimated standard errors is often higher if the $R^2$ of the scaling indicator is low. To produce Fig. 7, the percentage of trimmed replications is generally small (e.g., no more than $1.7 \%$ are trimmed when $n=200$ and no more than $0.3 \%$ are trimmed when $n=400$).

**Fig. 8:**
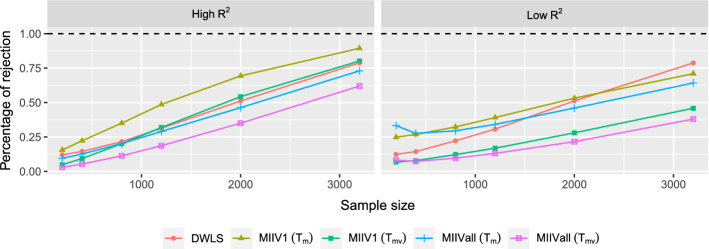
Percentages of rejection of the goodness-of-fit tests when the model is misspecified. The significance level is 0.05.

The empirical powers of the goodness-of-fit tests for the full model are in Fig. 8. Recall that the mean and variance adjusted test statistics ($T_{\mathrm{mv}}$) for MIIV1 and MIIVall had the most accurate Type I error probabilities in the correctly specified model under both the high and low $R^2$ conditions. The $T_\mathrm{m}$ test statistic from MIIV1 and MIIVall and the DWLS test statistic rejected too frequently with the correct model. For the misspecified model, using a strong scaling indicator tends to yield higher power than using a weak scaling indicator for all MIIV and DWLS test statistics. Nevertheless as *n* increases, the power of all tests tends to increase. Under the high $R^2$ condition, MIIV1 ($T_\mathrm{m}$) has the highest power with the test statistics MIIV1 ($T_{\mathrm{mv}}$), DWLS, and MIIVall ($T_\mathrm{m}$) the next highest. MIIVall ($T_{\mathrm{mv}}$) has the lowest power. Under the low $R^2$ condition, MIIV1 ($T_\mathrm{m}$), MIIVall ($T_\mathrm{m}$), and DWLS have the highest power followed by MIIV1 ($T_{\mathrm{mv}}$) and MIIVall ($T_{\mathrm{mv}}$). If we want the best combination of accurate Type I error and high statistical power under the high $R^2$ condition, then MIIV1 ($T_{\mathrm{mv}}$) is the best choice. The situation is more ambiguous for the low $R^2$ condition.

**Fig. 9:**
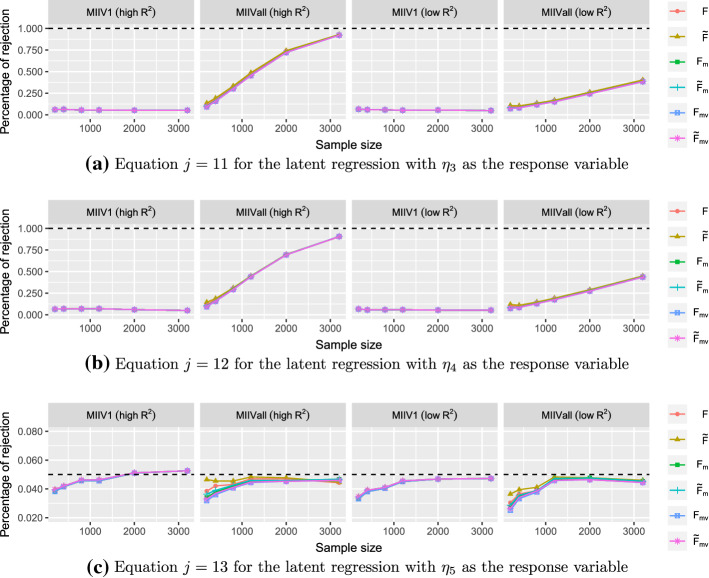
Overidentification test for the $\eta _3$ ($j=11$), $\eta _4$ ($j=12$), and $\eta _5$ ($j=13$) equations in the latent variable model at the significance level 0.05.

The MIIV estimators also provide overidentification tests for equations, one for each overidentified equation. Due to space limitaion, we only present the overidentification tests for the $\eta _3$ ($j=11$), $\eta _4$ ($j=12$), and $\eta _5$ ($j=13$) equations in the latent variable model. As discussed previously, we expect the empirical percentage rejection to approach 1 as *n* grows for equations $\eta _3$ ($j=11$) and $\eta _4$ ($j=12$) for MIIVall and to approach the nominal level 0.05 for $\eta _5$ ($j=13$) . Figure 9 illustrates that the sizes of all proposed overidentification tests become closer to the significance level 0.05 for the correctly specified $\eta _5$ equation with the rate of convergence more rapid if the $R^2$ of the scaling indicator is high. The power of the MIIVall tests for the $\eta _3$ ($j=11$) and $\eta _4$ ($j=12$) equations increase as *n* increases. However, the power of the MIIV1 tests is still far from 1, which is in line with the results in Jin and Cao (2018). It is also seen that the MIIV1 tests of *F*, $F_\mathrm{m}$, and $F_{\mathrm{mv}}$ perform almost the same and that the MIIV1 tests of ${\tilde{F}}$, ${\tilde{F}}_\mathrm{m}$, and ${\tilde{F}}_{\mathrm{mv}}$ perform almost the same. Hence, they are almost visually indistinguishable in the figure. For MIIVall, using a strong scaling indicator yields a higher power than using a weak scaling indicator.

## Empirical Example

**Fig. 10:**
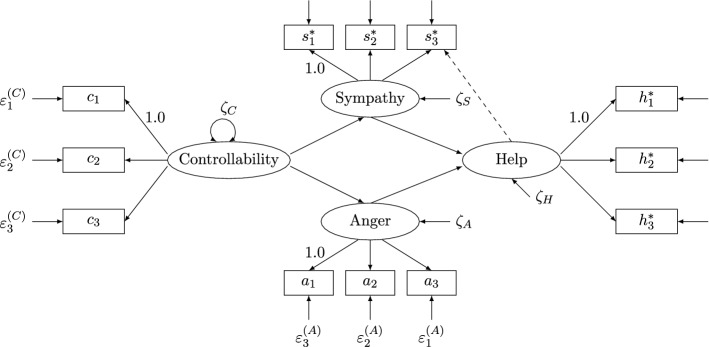
Path diagonal of the the Reisenzein (1986) dataset.

To demonstrate the use of our MIIV estimator, we consider the Reisenzein (1986) dataset with 138 observations that is available in the MIIVsem package. It is hypothesized that the effect of Controllability (perceived controllability over a situation) on Help (decision to help another person) is mediated by Sympathy and Anger, shown in Fig. 10. Each factor is measured by three indicators. Every observed variable of Controllability and Anger has nine categories and are treated as continuous. The observed variables of Sympathy and Help are measured by either the five-point or nine-point Likert scales. They are converted to five-point Likert scales and are treated as categorical.

We first use MIIV1, MIIVall and DWLS to estimate the model in Fig. 10 without the cross-loading $s_3^*$ on Help. For both MIIV1 and MIIVall, there exists eight indicator equations with factor loadings (with left-hand side variables $c_2$, $c_3$, $s_2^*$, $s_3^*$, $a_2$, $a_3^*$, $h_2^*$, $h_3^*$) and three latent variable equations with regression coefficients (with left-hand side variables Sympathy, Anger, and Help). It is seen from the top panel of Table 2 that all goodness-of-fit tests reject the null hypothesis that the model fits the data well. However, the robust CFI, TLI, and RMSEA with DWLS are 0.996, 0.995, and 0.053, indicating a reasonable fit. In contrast, Table 3 shows that the MIIVall specification tests are highly significant when used to test the equations for $s_3^*$ and Help. Here, the Bonferroni correction is used for simplicity to adjust the effect of multiple testing. Hence, the equations can be misspecified leading to invalid MIIVs. If we add the cross-loading $s_3^*$ on Help, all goodness-of-fit tests become insignificant (bottom panel of Table 2). It is seen from Table 4 that MIIV and DWLS yield similar point estimates and standard errors when the revised model is fitted.

**Table 2:** Goodness-of-fit tests of the Reisenzein (1986) data set.

Test statistic	MIIV1			MIIVall			DWLS		
	Value	df	*p* value	Value	df	*p* value	Value	df	*p* value
Without the cross loading ($s_{3}^{*}$ on *Help*)									
Mean	146.72	50.00	<0.0001	140.53	50.00	<0.0001	164.26	50.00	<0.0001
Mean-Var	39.45	13.44	<0.0001	42.78	15.22	<0.0001	51.09	15.55	<0.0001
With the cross loading ($s_{3}^{*}$ on *Help*)									
Mean	36.54	49.00	0.91	48.48	49.00	0.49	44.09	49.00	0.67
Mean-Var	10.39	13.93	0.73	14.00	14.15	0.46	16.17	17.97	0.58

*Mean* Mean-scaled test, *Mean-Var* Mean-variance adjusted test.

**Table 3:** Overidentification tests of the Reisenzein (1986) data set without the cross-loading $s_3^*$ on Help. The significant tests after the Bonferroni correction is boldfaced.

Test	Content	Left-hand side variable in each equation										
		$c_2$	$c_3$	$s_2^*$	$s_3^*$	$a_2$	$a_3$	$h_2^*$	$h_3^*$	*Sympathy*	*Anger*	*Help*
*F*	Value	17.48	6.83	12.56	55.68	9.79	15.03	8.33	15.27	10.34	14.43	**19**.**91**
	df	9.00	9.00	9.00	9.00	9.00	9.00	9.00	9.00	4.00	4.00	**5**.**00**
	p_Value	0.04	0.65	0.18	0.00	0.37	0.09	0.50	0.08	0.04	0.01	**0**.**00**
${\tilde{F}}$	Value	20.02	7.19	13.82	93.34	10.54	16.87	8.86	17.18	11.18	**16**.**12**	**23**.**26**
	df	9.00	9.00	9.00	9.00	9.00	9.00	9.00	9.00	4.00	**4**.**00**	**5**.**00**
	p_Value	0.02	0.62	0.13	0.00	0.31	0.05	0.45	0.05	0.02	**0**.**00**	**0**.**00**
$F_{m}$	Value	12.51	11.32	15.49	29.25	3.43	15.50	10.74	9.78	9.64	11.37	**18**.**24**
	df	9.00	9.00	9.00	9.00	9.00	9.00	9.00	9.00	4.00	4.00	**5**.**00**
	p_Value	0.19	0.25	0.08	0.00	0.94	0.08	0.29	0.37	0.05	0.02	**0**.**00**
${\tilde{F}}_{m}$	Value	12.64	11.42	15.69	29.96	3.44	15.70	10.84	9.86	9.81	11.61	**18**.**73**
	df	9.00	9.00	9.00	9.00	9.00	9.00	9.00	9.00	4.00	4.00	**5**.**00**
	p_Value	0.18	0.25	0.07	0.00	0.94	0.07	0.29	0.36	0.04	0.02	**0**.**00**
$F_{mv}$	Value	9.33	8.00	12.86	20.63	2.19	9.74	8.22	8.06	8.76	10.24	**16**.**50**
	df	6.71	6.36	7.47	6.35	5.75	5.66	6.89	7.42	3.63	3.60	**4**.**53**
	p_Value	0.21	0.27	0.09	0.00	0.88	0.12	0.30	0.37	0.05	0.03	**0**.**00**
${\tilde{F}}_{mv}$	Value	9.36	8.13	13.08	20.55	2.19	9.82	8.31	8.06	8.87	10.37	**16**.**91**
	df	6.67	6.41	7.50	6.17	5.73	5.63	6.90	7.36	3.62	3.57	**4**.**51**
	p_Value	0.20	0.26	0.09	0.00	0.88	0.11	0.30	0.36	0.05	0.03	**0**.**00**

**Table 4:** Point estimates and the standard errors of the Reisenzein (1986) dataset with the cross-loading $s_3^*$ on Help. For MIIV, the standard error of $\hat{{\varvec{\theta }}_1}$ are computed from (9).

Parameter	DWLS		MIIV1		MIIVall	
	Est.	SE	Est.	SE	Est.	SE
Parameters in ${\varvec{\theta }}_{1}$						
Controllability$\rightarrow c_{2}$	1.089	0.193	1.088	0.154	1.042	0.153
Controllability$\rightarrow c_{3}$	1.275	0.262	1.169	0.188	1.149	0.188
Sympathy$\rightarrow s_{2}^*$	0.761	0.060	0.756	0.054	0.734	0.056
Sympathy$\rightarrow s_{3}^*$	0.603	0.058	0.603	0.077	0.555	0.076
Anger$\rightarrow a_{2}$	0.933	0.154	0.909	0.128	0.906	0.134
Anger$\rightarrow a_{3}$	0.885	0.130	0.881	0.101	0.882	0.116
Help$\rightarrow h_{2}^*$	0.951	0.031	0.953	0.032	0.955	0.033
Help$\rightarrow h_{3}^*$	0.806	0.040	0.819	0.040	0.829	0.038
Help$\rightarrow s_{3}^*$	0.339	0.054	0.338	0.064	0.361	0.065
Controllability$\rightarrow$Sympathy	$-$0.278	0.053	$-$0.267	0.044	$-$0.279	0.045
Controllability$\rightarrow$Anger	0.651	0.149	0.652	0.133	0.636	0.132
Sympathy$\rightarrow$Help	0.265	0.081	0.340	0.096	0.360	0.104
Anger$\rightarrow$Help	$-$0.243	0.050	$-$0.212	0.048	$-$0.199	0.048
Parameters in ${\varvec{\theta }}_{2}$						
$\text {var}\left( \zeta _{C}\right)$	4.965	1.699	5.132	1.534	5.206	1.599
$\text {var}\left( \zeta _{S}\right)$	0.610	0.102	0.601	0.083	0.590	0.086
$\text {var}\left( \zeta _{A}\right)$	1.981	0.596	2.144	0.621	2.293	0.650
$\text {var}\left( \zeta _{H}\right)$	0.524	0.071	0.507	0.075	0.500	0.075
$\text {var}\left( \varepsilon _{1}^{(C)}\right)$	2.103	0.579	1.935	0.350	1.861	0.315
$\text {var}\left( \varepsilon _{2}^{(C)}\right)$	2.104	0.734	1.918	0.402	2.333	0.424
$\text {var}\left( \varepsilon _{3}^{(C)}\right)$	0.824	0.858	1.881	0.346	2.021	0.484
$\text {var}\left( \varepsilon _{1}^{(A)}\right)$	1.442	0.402	1.201	0.326	1.129	0.328
$\text {var}\left( \varepsilon _{2}^{(A)}\right)$	1.730	0.389	1.717	0.276	1.678	0.269
$\text {var}\left( \varepsilon _{3}^{(A)}\right)$	1.739	0.385	1.582	0.309	1.520	0.330

*Est.* Estimate, *SE* Standard error.

## Discussion and Conclusion

We proposed a unified MIIV approach that handles a mixture of continuous, ordinal, or binary observed endogenous variables in SEM. Our method only requires a consistent estimator of the covariance matrix and an asymptotic covariance matrix of its elements. We provide point estimators of all parameters and their asymptotic standard errors. Furthermore, we provide model goodness of fit test statistics and local tests of overidentified equations. The latter of which correspond to the classic Sargan’s Chi-square test and the tests in Jin and Cao (2018). In addition, we give Satorra–Bentler-type modifications to these test statistics. The simulation study shows that the small sample properties of the proposed MIIV approach is generally in line with the theoretical results. That is, the MIIV estimators are more robust to structural misspecifications than are the systemwide estimators; the overidentification equation tests provide useful local tests of fit, and the model goodness of fit tests provide useful diagnostics on global fit. The performance of these MIIV tests is best with strong MIIVs and deteriorates if the MIIVs are weak.

The MIIV procedure is applicable to a large class of latent variable models, as long as they can be expressed as (1) and (2). Among these are the confirmatory factor analysis model (Jin et al., 2016; Jin and Cao, 2018; Nestler, 2013), the latent growth model (Nestler, 2015b), and specification tests for nonlinear terms in the latent variable model (Nestler, 2015a). The MIIV estimation essentially depends on ${\varvec{S}}$, which can be interpreted as a method of moment. Various latent variable models such as the item response theory models are expressed from the likelihood perspective. When using the probit link, some connections between the MIIV estimates and item response theory parameters are possible (Takane and de Leeuw, 1987). But for item response-type models in general the connections to a MIIV approach are less clear.

The finite sample equivalence among various test statistics depend on the estimator of ${\varvec{\Omega }}$. The estimators $\hat{{\varvec{\Omega }}}$ and $\tilde{{\varvec{\Omega }}}$ are asymptotically the same but differ by a consistent estimator of ${\varvec{0}}$ if all MIIVs are valid. If some MIIVs are not valid, $\hat{{\varvec{\Omega }}}$ and $\tilde{{\varvec{\Omega }}}$ are not always asymptotically the same. In other words, the established equivalence is only for the null distribution. A rigorous power analysis and extensive simulation studies to investigate the distributions of the test statistics under the alternative hypothesis and the effects of using different ${\varvec{\Omega }}$ estimators in order to make further recommendations.

We also explored weak instruments in our simulation. An IV is commonly viewed as weak if its correlation with the endogenous variable is small. With the presence of weak IVs, it is known in the econometrics literature that the 2SLS estimator is biased, and the overidentification test has the wrong size when even a small correlation between instruments and equation errors exist (e.g., Bound et al., 1995; Hahn and Hausman, 2003; 2005). In our simulation, we considered both MIIV1 and MIIVall. The weak IVs are not excluded from MIIVall, but MIIV1 only incorporates the strongest IVs for the given data set. We found that scaling indicators with low $R^2$s were associated with weak instruments. A number of authors have proposed diagnostics for weak instruments including Hahn and Hausman (2002), Kleibergen and Paap (2006), Olea and Pflueger (2013), Shea (1997), and Stock and Yogo (2005). Fisher and Bollen (2020) showed that the accuracy of estimation is negatively related with the Shea’s partial $R^2$. Further studies are needed to provide guidelines on what to do when all IVs are weak or when some IVs are strong but others are weak. Finally, we recognize the limits of any simulation study and encourage other simulation studies that explore a wider variety of conditions to determine the degree to which our findings generalize.

### Supplementary Information

Below is the link to the electronic supplementary material.

Supplementary material 1 (gz 37 KB)

## Appendix: Mathematical Proof

### Proof of Proposition 1

Let $\sigma _{\mathrm{vz}}$ be an element in ${\varvec{\Sigma }}_{\mathrm{vz},j}$, $\sigma _{\mathrm{vv}}$ be an element in ${\varvec{\Sigma }}_{\mathrm{vv},j}$, and $\sigma _{\mathrm{vy}}$ be an element in ${\varvec{\Sigma }}_{\mathrm{vy},j}$. Then

$$\begin{aligned}&\frac{\partial {\varvec{g}}_{j}\left( {\varvec{\sigma }}\right) }{\partial \sigma _{\mathrm{vz}}}=-\frac{\partial {\varvec{\Sigma }}_{\mathrm{vz},j}}{\partial \sigma _{\mathrm{vz}}}{\varvec{\theta }}_{1}^{(j)},\quad \frac{\partial {\varvec{g}}_{j}\left( {\varvec{\sigma }}\right) }{\partial \sigma _{vv}}={\varvec{0}},\quad \frac{\partial {\varvec{g}}_{j}\left( {\varvec{\sigma }}\right) }{\partial \sigma _{vy}}=\frac{\partial {\varvec{\Sigma }}_{vy,j}}{\partial \sigma _{vy}}. \end{aligned}$$

For any other $\sigma$ that does not belong to ${\varvec{\Sigma }}_{\mathrm{vz},j}$, ${\varvec{\Sigma }}_{\mathrm{vv},j}$, nor ${\varvec{\Sigma }}_{\mathrm{vy},j}$, $\partial {\varvec{g}}_{j}\left( {\varvec{\sigma }}\right) /\partial \sigma =0$. In matrix form, the nonzero part in $\partial {\varvec{g}}_{j}\left( {\varvec{\sigma }}\right) /\partial {\varvec{{\varvec{\sigma }}}}^{T}$ is essentially

$$\begin{aligned}&\begin{pmatrix}{\varvec{I}}&\,&-{\varvec{\theta }}_{1}^{(j)T}\otimes {\varvec{I}}\end{pmatrix}, \end{aligned}$$

subject to a permutation of columns, where $\otimes$ is the Kronecker product. If $\hat{{\varvec{\Upsilon }}}=2{\varvec{D}}^{+}\left( {\varvec{S}}\otimes {\varvec{S}}\right) {\varvec{D}}^{+T}$, the estimator of ${\varvec{\Upsilon }}$ that corresponds to the nonzero part in $\partial {\varvec{g}}_{j}\left( {\varvec{s}}\right) /\partial {\varvec{{\varvec{\sigma }}}}^{T}$ is

$$\begin{aligned} \begin{pmatrix}{\varvec{S}}_{\mathrm{yy},j}{\varvec{S}}_{\mathrm{vv},j} &{} &{} {\varvec{S}}_{\mathrm{zy},j}^{T}\otimes {\varvec{S}}_{\mathrm{vv},j}\\ {\varvec{S}}_{\mathrm{zy},j}\otimes {\varvec{S}}_{\mathrm{vv},j} &{} &{} {\varvec{S}}_{\mathrm{zz},j}\otimes {\varvec{S}}_{\mathrm{vv},j} \end{pmatrix}+\begin{pmatrix}{\varvec{S}}_{\mathrm{vy},j}{\varvec{S}}_{\mathrm{vy},j}^{T} &{} &{} {\varvec{S}}_{\mathrm{vz},j}\otimes {\varvec{S}}_{\mathrm{vy},j}^{T}\\ {\varvec{S}}_{\mathrm{vz},j}^{T}\otimes {\varvec{S}}_{\mathrm{vu},j} &{} &{} \hat{{\varvec{A}}}_{j} \end{pmatrix}, \end{aligned}$$

where $\hat{{\varvec{A}}}_{j}$ is a block matrix such that the (*i*, *k*)th block is ${\varvec{S}}_{\mathrm{vz}_{k},j}{\varvec{S}}_{\mathrm{vz}_{i},j}^{T}$. Some algebra shows that

$$\begin{aligned} \frac{\partial {\varvec{g}}_{j}\left( {\varvec{s}}\right) }{\partial {\varvec{\sigma }}^{T}}\hat{{\varvec{\Upsilon }}}\left( \frac{\partial {\varvec{g}}_{j}\left( {\varvec{s}}\right) }{\partial {\varvec{\sigma }}^{T}}\right) ^{T}=&\left( {\varvec{S}}_{\mathrm{yy},j}-2\hat{{\varvec{\theta }}}_{1}^{(j)T}{\varvec{S}}_{\mathrm{zy},j}+\hat{{\varvec{\theta }}}_{1}^{(j)T}{\varvec{S}}_{\mathrm{zz},j}\hat{{\varvec{\theta }}}_{1}^{(j)}\right) {\varvec{S}}_{\mathrm{vv},j}+{\varvec{S}}_{\mathrm{vy},j}{\varvec{S}}_{\mathrm{vy},j}^{T}\\&-\left( {\varvec{S}}_{\mathrm{vz},j}\hat{{\varvec{\theta }}}_{1}^{(j)}\right) \otimes {\varvec{S}}_{\mathrm{vy},j}^{T}-\left( \hat{{\varvec{\theta }}}_{1}^{(j)T}{\varvec{S}}_{\mathrm{vz},j}^{T}\right) \otimes {\varvec{S}}_{\mathrm{vy},j} \\&+\left( \hat{{\varvec{\theta }}}_{1}^{(j)T}\otimes {\varvec{I}}\right) \hat{{\varvec{A}}}_{j}\left( \hat{{\varvec{\theta }}}_{1}^{(j)}\otimes {\varvec{I}}\right) \\ =&{\hat{\varphi }}_{j}^{2}{\varvec{S}}_{\mathrm{vv},j}+\left( {\varvec{S}}_{\mathrm{vy},j}-{\varvec{S}}_{\mathrm{vz},j}\hat{{\varvec{\theta }}}_{1}^{(j)}\right) \left( {\varvec{S}}_{\mathrm{vy},j}-{\varvec{S}}_{\mathrm{vz},j}\hat{{\varvec{\theta }}}_{1}^{(j)}\right) ^{T}, \end{aligned}$$

where the last equality holds because of

$$\begin{aligned} \left( \hat{{\varvec{\theta }}}_{1}^{(j)T}\otimes {\varvec{I}}\right) \hat{{\varvec{A}}}_{j}\left( \hat{{\varvec{\theta }}}_{1}^{(j)}\otimes {\varvec{I}}\right) ={\varvec{S}}_{vz,j}\hat{{\varvec{\theta }}}_{1}^{(j)}\hat{{\varvec{\theta }}}_{1}^{(j)T}{\varvec{S}}_{vz,j}^{T} \end{aligned}$$

by expanding the elements in the Kronecker product. Then, the proposition holds because

$$\begin{aligned} {\varvec{S}}_{vz,j}^{T}{\varvec{S}}_{vv,j}^{-1}\left( {\varvec{S}}_{vy,j}-{\varvec{S}}_{vz,j}\hat{{\varvec{\theta }}}_{1}^{(j)}\right) =&{\varvec{0}}. \end{aligned}$$

$$\square $$

### Proof of Proposition 2

From the proof of Proposition 1, the nonzero part in $\partial {\varvec{g}}_{j}\left( {\varvec{\sigma }}\right) /\partial {\varvec{{\varvec{\sigma }}}}^{T}$ is essentially

$$\begin{aligned}&\begin{pmatrix}{\varvec{I}}&\,&-{\varvec{\theta }}_1^{(j)T}\otimes {\varvec{I}}\end{pmatrix}, \end{aligned}$$

subject to a permutation of columns. If all observed variables are continuous, the submatrix in ${\varvec{\Upsilon }}$ corresponding to ${\varvec{\Sigma }}_{vy,j}$ and ${\varvec{\Sigma }}_{vz,j}$ is of the form

$$\begin{aligned} \begin{pmatrix} E\left( y_{j}^{*2}{\varvec{v}}_{j}^{*}{\varvec{v}}_{j}^{*T}\right) -E\left( y_{j}^{*}{\varvec{v}}_{j}^{*}\right) E\left( y_{j}^{*}{\varvec{v}}_{j}^{*T}\right) &{} E\left[ \left( y_{j}^{*}{\varvec{z}}_{j}^{*T}\right) \otimes \left( {\varvec{v}}_{j}^{*}{\varvec{v}}_{j}^{*T}\right) \right] -E\left( y_{j}^{*}\otimes {\varvec{v}}_{j}^{*}\right) E\left( {\varvec{z}}_{j}^{*T}\otimes {\varvec{v}}_{j}^{*T}\right) \\ E\left[ \left( y_{j}^{*} {\varvec{z}}_{j}^{*}\right) \otimes \left( {\varvec{v}}_{j}^{*}{\varvec{v}}_{j}^{*T}\right) \right] -E\left( {\varvec{z}}_{j}^{*}\otimes {\varvec{v}}_{j}^{*}\right) E\left( y_{j}^{*}\otimes {\varvec{v}}_{j}^{*T}\right) &{} E\left[ \left( {\varvec{z}}_{j}^{*}{\varvec{z}}_{j}^{*T}\right) \otimes \left( {\varvec{v}}_{j}^{*}{\varvec{v}}_{j}^{*T}\right) \right] -E\left( {\varvec{z}}_{j}^{*}\otimes {\varvec{v}}_{j}^{*}\right) E\left( {\varvec{z}}_{j}^{*T}\otimes {\varvec{v}}_{j}^{*T}\right) \end{pmatrix}. \end{aligned}$$

Some algebra shows that

$$\begin{aligned} \frac{\partial {\varvec{g}}_{j}\left( {\varvec{\sigma }}\right) }{\partial {\varvec{\sigma }}^{T}}{\varvec{\Upsilon }}\left( \frac{\partial {\varvec{g}}_{j}\left( {\varvec{\sigma }}\right) }{\partial {\varvec{\sigma }}^{T}}\right) ^{T}= & {} E\left( y_j^{*2}{\varvec{v}}_{j}^{*}{\varvec{v}}_{j}^{*T}\right) -2E\left[ y_{j}^{*}\left( {\varvec{z}}_{j}^{*T}{\varvec{\theta }}_{1}^{(j)}\right) \left( {\varvec{v}}_{j}^{*}{\varvec{v}}_{j}^{*T}\right) \right] \\&+E\left[ \left( {\varvec{z}}_{j}^{*T}{\varvec{\theta }}_{1}^{(j)}\right) ^{2}\left( {\varvec{v}}_{j}^{*}{\varvec{v}}_{j}^{*T}\right) \right] \\&-\left( {\varvec{\Sigma }}_{vy,j}-{\varvec{\Sigma }}_{vz,j}{\varvec{\theta }}_{1}^{(j)}\right) \left( {\varvec{\Sigma }}_{vy,j}-{\varvec{\Sigma }}_{vz,j}{\varvec{\theta }}_{1}^{(j)}\right) ^{T}\\= & {} E\left( {\varvec{v}}_{j}^{*}{\varvec{v}}_{j}^{*T}e_{j}^{2}\right) -\left( {\varvec{\Sigma }}_{vy,j}-{\varvec{\Sigma }}_{vz,j}{\varvec{\theta }}_{1}^{(j)}\right) \left( {\varvec{\Sigma }}_{vy,j}-{\varvec{\Sigma }}_{vz,j}{\varvec{\theta }}_{1}^{(j)}\right) ^{T}. \end{aligned}$$

If the model is correctly specified and all MIIVs are valid, then $E\left( e_{j}^{2} {\varvec{v}}_{j}^{*} {\varvec{v}}_{j}^{*T}\right) =\varphi _{j}^{2}{\varvec{\Sigma }}_{vv,j}$ and ${\varvec{\Sigma }}_{vy,j}-{\varvec{\Sigma }}_{vz,j}{\varvec{\theta }}_{1}^{(j)}={\varvec{0}}$, which completes the proof. $\square$

### Proof of Equation (14)

The assumptions that we need include (1) $\hat{{\varvec{\theta }}}\overset{p}{\rightarrow }{\varvec{\theta }}$, (2) ${\varvec{s}}-{\varvec{\sigma }}\left( \hat{{\varvec{\theta }}}\right) \overset{p}{\rightarrow }{\varvec{0}}$, (3) $\partial ^{3}T\left( {\varvec{\theta }}\right) /\partial \theta _{2,j}\partial {\varvec{\theta }}_{2}\partial {\varvec{\theta }}_{2}^{T}$ is bounded in probability in an open set ${\mathbb {O}}$ that contains ${\varvec{\theta }}_2$ for any $\theta _{2,j}$, the *j*th entry in ${\varvec{\theta }}_{2}$, and (4) $\partial ^{2}{\varvec{\sigma }}\left( {\varvec{\theta }}\right) /\partial {\varvec{\theta }}_{2}\partial {\varvec{\theta }}^{T}$ is also bounded in ${\mathbb {O}}$. Assumptions (1) and (2) hold if the model is correctly specified and all MIIVs are valid. Assumptions (3) and (4) hold since $T\left( {\varvec{\theta }}\right)$ and ${\varvec{\sigma }}\left( {\varvec{\theta }}\right)$ have continuous higher-order partial derivatives given by our SEM model.

Since ${\varvec{\sigma }}\left( {\varvec{\theta }}\right)$ has continuous third-order partial derivatives with respect to ${\varvec{\theta }}$, the standard Taylor expansion yields

25 $$\begin{aligned} 0= & {} \frac{\partial T\left( \hat{{\varvec{\theta }}}_{1},\hat{{\varvec{\theta }}}_{2}\right) }{\partial \theta _{2,j}}\nonumber \\= & {} \frac{\partial T\left( \hat{{\varvec{\theta }}}_{1},{\varvec{\theta }}_{2}\right) }{\partial \theta _{2,j}}+\frac{\partial ^{2}T\left( \hat{{\varvec{\theta }}}_{1},{\varvec{\theta }}_{2}\right) }{\partial \theta _{2,j}\partial {\varvec{\theta }}_{2}^{T}}\left( \hat{{\varvec{\theta }}}_{2}-{\varvec{\theta }}_{2}\right) \nonumber \\&+\frac{1}{2}\left( \hat{{\varvec{\theta }}}_{2}-{\varvec{\theta }}_{2}\right) ^{T}\frac{\partial ^{3}T\left( \hat{{\varvec{\theta }}}_{1},\tilde{{\varvec{\theta }}}_{2}\right) }{\partial \theta _{2,j}\partial {\varvec{\theta }}_{2}\partial {\varvec{\theta }}_{2}^{T}}\left( \hat{{\varvec{\theta }}}_{2}-{\varvec{\theta }}_{2}\right) , \end{aligned}$$

where $\theta _{2,j}$ is the *j*th entry of ${\varvec{\theta }}_{2}$ and $\tilde{{\varvec{\theta }}}_{2}$ lies between $\hat{{\varvec{\theta }}}_{2}$ and ${\varvec{\theta }}_{2}$. Similarly, the standard Taylor expansion yields

26 $$\begin{aligned} \frac{\partial T\left( \hat{{\varvec{\theta }}}_{1},{\varvec{\theta }}_{2}\right) }{\partial \theta _{2,j}}= & {} \frac{\partial T\left( {\varvec{\theta }}\right) }{\partial \theta _{2,j}}+\frac{\partial ^{2}T\left( {\varvec{\theta }}\right) }{\partial \theta _{2,j}\partial {\varvec{\theta }}_{1}^{T}}\left( \hat{{\varvec{\theta }}}_{1}-{\varvec{\theta }}_{1}\right) \nonumber \\&+\frac{1}{2}\left( \hat{{\varvec{\theta }}}_{1}-{\varvec{\theta }}_{1}\right) ^{T}\frac{\partial ^{3}T\left( \tilde{{\varvec{\theta }}}_{1},{\varvec{\theta }}_{2}\right) }{\partial \theta _{2,j}\partial {\varvec{\theta }}_{1}\partial {\varvec{\theta }}_{1}^{T}}\left( \hat{{\varvec{\theta }}}_{1}-{\varvec{\theta }}_{1}\right) , \end{aligned}$$

and

27 $$\begin{aligned} \frac{\partial ^{2}T\left( \hat{{\varvec{\theta }}}_{1},{\varvec{\theta }}_{2}\right) }{\partial \theta _{2,j}\partial {\varvec{\theta }}_{2}}= & {} \frac{\partial ^{2}T\left( {\varvec{\theta }}\right) }{\partial \theta _{2,j}\partial {\varvec{\theta }}_{2}}+\frac{\partial ^{3}T\left( \check{{\varvec{\theta }}}_{1},{\varvec{\theta }}_{2}\right) }{\partial \theta _{2,j}\partial {\varvec{\theta }}_{1}\partial {\varvec{\theta }}_{2}^{T}}\left( \hat{{\varvec{\theta }}}_{1}-{\varvec{\theta }}_{1}\right) , \end{aligned}$$

where both $\tilde{{\varvec{\theta }}}_{1}$ and $\check{{\varvec{\theta }}}_{1}$ lie between $\hat{{\varvec{\theta }}}_{1}$ and ${\varvec{\theta }}_{1}$. Then, after plugging (26) and (27) into (25), we obtain

$$\begin{aligned}&\sqrt{n}\left[ \frac{\partial ^{2}T\left( {\varvec{\theta }}\right) }{\partial \theta _{2,j}\partial {\varvec{\theta }}_{2}^{T}}+\left( \hat{{\varvec{\theta }}}_{1}-{\varvec{\theta }}_{1}\right) ^{T}\frac{\partial ^{3}T\left( \check{{\varvec{\theta }}}_{1},{\varvec{\theta }}_{2}\right) }{\partial \theta _{2,j}\partial {\varvec{\theta }}_{1}\partial {\varvec{\theta }}_{2}^{T}}+\frac{1}{2}\left( \hat{{\varvec{\theta }}}_{2}-{\varvec{\theta }}_{2}\right) ^{T}\frac{\partial ^{3}T\left( \hat{{\varvec{\theta }}}_{1},\tilde{{\varvec{\theta }}}_{2}\right) }{\partial \theta _{2,j}\partial {\varvec{\theta }}_{2}\partial {\varvec{\theta }}_{2}^{T}}\right] \left( \hat{{\varvec{\theta }}}_{2}-{\varvec{\theta }}_{2}\right) \\&\quad = -\sqrt{n}\frac{\partial T\left( {\varvec{\theta }}\right) }{\partial \theta _{2,j}}-\frac{\partial ^{2}T\left( {\varvec{\theta }}\right) }{\partial \theta _{2,j}\partial {\varvec{\theta }}_{1}^{T}}\sqrt{n}\left( \hat{{\varvec{\theta }}}_{1}-{\varvec{\theta }}_{1}\right) -\frac{1}{2}\sqrt{n}\left( \hat{{\varvec{\theta }}}_{1}-{\varvec{\theta }}_{1}\right) ^{T}\frac{\partial ^{3}T\left( \tilde{{\varvec{\theta }}}_{1},{\varvec{\theta }}_{2}\right) }{\partial \theta _{2,j}\partial {\varvec{\theta }}_{1}\partial {\varvec{\theta }}_{1}^{T}}\left( \hat{{\varvec{\theta }}}_{1}-{\varvec{\theta }}_{1}\right) . \end{aligned}$$

By the assumptions mentioned in the beginning of the proof and $\hat{{\varvec{W}}}\overset{p}{\rightarrow }{\varvec{W}}$, we obtain

$$\begin{aligned} \left( \hat{{\varvec{\theta }}}_{1}-{\varvec{\theta }}_{1}\right) ^{T}\frac{\partial ^{3}T\left( \check{{\varvec{\theta }}}_{1},{\varvec{\theta }}_{2}\right) }{\partial \theta _{2,j}\partial {\varvec{\theta }}_{1}\partial {\varvec{\theta }}_{2}^{T}}&\overset{p}{\rightarrow }&0,\\ \left( \hat{{\varvec{\theta }}}_{2}-{\varvec{\theta }}_{2}\right) ^{T}\frac{\partial ^{3}T\left( \hat{{\varvec{\theta }}}_{1},\tilde{{\varvec{\theta }}}_{2}\right) }{\partial \theta _{2,j}\partial {\varvec{\theta }}_{2}\partial {\varvec{\theta }}_{2}^{T}}&\overset{p}{\rightarrow }&0,\\ \left( \hat{{\varvec{\theta }}}_{1}-{\varvec{\theta }}_{1}\right) ^{T}\frac{\partial ^{3}T\left( \tilde{{\varvec{\theta }}}_{1},{\varvec{\theta }}_{2}\right) }{\partial \theta _{2,j}\partial {\varvec{\theta }}_{1}\partial {\varvec{\theta }}_{1}^{T}}\left( \hat{{\varvec{\theta }}}_{1}-{\varvec{\theta }}_{1}\right)&\overset{p}{\rightarrow }&0. \end{aligned}$$

Hence,

$$\begin{aligned} \frac{\partial ^{2}T\left( {\varvec{\theta }}\right) }{\partial \theta _{2,j}\partial {\varvec{\theta }}_{2}^{T}}\sqrt{n}\left( \hat{{\varvec{\theta }}}_{2}-{\varvec{\theta }}_{2}\right)= & {} -\sqrt{n}\frac{\partial T\left( {\varvec{\theta }}\right) }{\partial \theta _{2,j}}-\frac{\partial ^{2}T\left( {\varvec{\theta }}\right) }{\partial \theta _{2,j}\partial {\varvec{\theta }}_{1}^{T}}\sqrt{n}\left( \hat{{\varvec{\theta }}}_{1}-{\varvec{\theta }}_{1}\right) +o_{\text {P}}\left( 1\right) . \end{aligned}$$

By collecting all entries in ${\varvec{\theta }}_{2}$, we obtain

$$\begin{aligned} \frac{\partial ^{2}T\left( {\varvec{\theta }}\right) }{\partial {\varvec{\theta }}_{2}\partial {\varvec{\theta }}_{2}^{T}}\sqrt{n}\left( \hat{{\varvec{\theta }}}_{2}-{\varvec{\theta }}_{2}\right)= & {} -\sqrt{n}\frac{\partial T\left( {\varvec{\theta }}\right) }{\partial {\varvec{\theta }}_{2}}-\frac{\partial ^{2}T\left( {\varvec{\theta }}\right) }{\partial {\varvec{\theta }}_{2}\partial {\varvec{\theta }}_{1}^{T}}\sqrt{n}\left( \hat{{\varvec{\theta }}}_{1}-{\varvec{\theta }}_{1}\right) +o_{\text {P}}\left( 1\right) . \end{aligned}$$

By equation (8), we further obtain

28 $$\begin{aligned} \frac{\partial ^{2}T\left( {\varvec{\theta }}\right) }{\partial {\varvec{\theta }}_{2}\partial {\varvec{\theta }}_{2}^{T}}\sqrt{n}\left( \hat{{\varvec{\theta }}}_{2}-{\varvec{\theta }}_{2}\right)= & {} -\sqrt{n}\frac{\partial T\left( {\varvec{\theta }}\right) }{\partial {\varvec{\theta }}_{2}}-\frac{\partial ^{2}T\left( {\varvec{\theta }}\right) }{\partial {\varvec{\theta }}_{2}\partial {\varvec{\theta }}_{1}^{T}}{\varvec{K}}\left( {\varvec{\sigma }}\right) \sqrt{n}\left( {\varvec{s}}-{\varvec{\sigma }}\right) +o_{\text {P}}\left( 1\right) . \end{aligned}$$

Note that the partial derivatives in (28) are

$$\begin{aligned} \frac{\partial T\left( {\varvec{\theta }}\right) }{\partial {\varvec{\theta }}_{2}}= & {} -2{\varvec{J}}_{2}^{T}\left( {\varvec{\theta }}\right) \hat{{\varvec{W}}}\left( {\varvec{s}}-{\varvec{\sigma }}\left( {\varvec{\theta }}\right) \right) ,\\ \frac{\partial ^{2}T\left( {\varvec{\theta }}\right) }{\partial {\varvec{\theta }}_{2}\partial {\varvec{\theta }}_{1}^{T}}= & {} 2{\varvec{J}}_{2}^{T}\left( {\varvec{\theta }}\right) \hat{{\varvec{W}}}{\varvec{J}}_{1}\left( {\varvec{\theta }}\right) -2\frac{\partial ^{2}{\varvec{\sigma }}\left( {\varvec{\theta }}\right) }{\partial {\varvec{\theta }}_{2}\partial {\varvec{\theta }}_{1}^{T}}\left\{ {\varvec{I}}\otimes \left[ \hat{{\varvec{W}}}\left( {\varvec{s}}-{\varvec{\sigma }}\left( {\varvec{\theta }}\right) \right) \right] \right\} ,\\ \frac{\partial ^{2}T\left( {\varvec{\theta }}\right) }{\partial {\varvec{\theta }}_{2}\partial {\varvec{\theta }}_{2}^{T}}= & {} 2{\varvec{J}}_{2}^{T}\left( {\varvec{\theta }}\right) \hat{{\varvec{W}}}{\varvec{J}}_{2}\left( {\varvec{\theta }}\right) -2\frac{\partial ^{2}{\varvec{\sigma }}\left( {\varvec{\theta }}\right) }{\partial {\varvec{\theta }}_{2}\partial {\varvec{\theta }}_{2}^{T}}\left\{ {\varvec{I}}\otimes \left[ \hat{{\varvec{W}}}\left( {\varvec{s}}-{\varvec{\sigma }}\left( {\varvec{\theta }}\right) \right) \right] \right\} , \end{aligned}$$

where ${\varvec{s}}-{\varvec{\sigma }}\left( \hat{{\varvec{\theta }}}\right) \overset{p}{\rightarrow }{\varvec{0}}$ under our assumption. Then we get the expansion (14). $\square$

*Proof of the distribution of* $nT\left( \hat{{\varvec{\theta }}}\right)$. If the model is correctly specified, expansions (8) and (14) imply

$$\begin{aligned} \sqrt{n}\left( \hat{{\varvec{\theta }}}-{\varvec{\theta }}\right)= & {} \begin{pmatrix}{\varvec{K}}\left( {\varvec{\sigma }}\right) \\ {\varvec{C}}\left( {\varvec{\theta }},{\varvec{\sigma }}\right) \end{pmatrix}\sqrt{n}\left( {\varvec{s}}-{\varvec{\sigma }}\right) +o_{\text {P}}\left( 1\right) . \end{aligned}$$

The delta method implies

$$\begin{aligned} \sqrt{n}\left( {\varvec{\sigma }}\left( \hat{{\varvec{\theta }}}\right) -{\varvec{\sigma }}\right)= & {} {\varvec{J}}\left( {\varvec{\theta }}\right) \begin{pmatrix}{\varvec{K}}\left( {\varvec{\sigma }}\right) \\ {\varvec{C}}\left( {\varvec{\theta }},{\varvec{\sigma }}\right) \end{pmatrix}\sqrt{n}\left( {\varvec{s}}-{\varvec{\sigma }}\right) +o_{\text {P}}\left( 1\right) , \end{aligned}$$

where $${\varvec{J}}\left( {\varvec{\theta }}\right) =\partial {\varvec{\sigma }}\left( {\varvec{\theta }}\right) /\partial {\varvec{\theta }}^{T}$$. Hence,

$$\begin{aligned} \sqrt{n}\left( {\varvec{s}}-{\varvec{\sigma }}\left( \hat{{\varvec{\theta }}}\right) \right)= & {} \sqrt{n}\left( {\varvec{s}}-{\varvec{\sigma }}\right) -\sqrt{n}\left( {\varvec{\sigma }}\left( \hat{{\varvec{\theta }}}\right) -{\varvec{\sigma }}\right) \\= & {} \left[ {\varvec{I}}-{\varvec{J}}\left( {\varvec{\theta }}\right) \begin{pmatrix}{\varvec{K}}\left( {\varvec{\sigma }}\right) \\ {\varvec{C}}\left( {\varvec{\theta }},{\varvec{\sigma }}\right) \end{pmatrix}\right] \sqrt{n}\left( {\varvec{s}}-{\varvec{\sigma }}\right) +o_{\text {P}}\left( 1\right) . \end{aligned}$$

Then, $nT\left( \hat{{\varvec{\theta }}}\right)$ converges in distribution to a weighted sum of independent Chi-square random variables with 1 degree of freedom, where the weights are the eigenvalues of (16). $\square$

### Proof of Theorem 1

Theorem 2 in Styan (1970) indicates that the quadratic form is Chi-square distributed if and only if

29 $$\begin{aligned}&\left( {\varvec{Q}}{\varvec{Q}}^{T}\right) {\varvec{G}}\left( {\varvec{Q}}{\varvec{Q}}^{T}\right) {\varvec{G}}\left( {\varvec{Q}}{\varvec{Q}}^{T}\right) =\left( {\varvec{Q}}{\varvec{Q}}^{T}\right) {\varvec{G}}\left( {\varvec{Q}}{\varvec{Q}}^{T}\right) , \end{aligned}$$

30 $$\begin{aligned} \text {and }\quad&\text {rank}\left\{ \left( {\varvec{Q}}{\varvec{Q}}^{T}\right) {\varvec{G}}\left( {\varvec{Q}}{\varvec{Q}}^{T}\right) \right\} =\text {tr}\left\{ {\varvec{G}}\left( {\varvec{Q}}{\varvec{Q}}^{T}\right) \right\} , \end{aligned}$$

where $\text {rank}\left\{ \right\}$ and $\text {tr}\left\{ \right\}$ denote the rank and the trace of the enclosed matrix, respectively. Since ${\varvec{G}}$ is the Moore–Penrose inverse of ${\varvec{Q}}{\varvec{Q}}^{T}$, condition (29) is naturally satisfied. Hence, it suffices to show condition (30). It can be easily shown that ${\varvec{G}}\left( {\varvec{Q}}{\varvec{Q}}^{T}\right)$ is an idempotent matrix when ${\varvec{G}}$ is a Moore–Penrose inverse. Hence,

$$\begin{aligned} \text {tr}\left\{ {\varvec{G}}\left( {\varvec{Q}}{\varvec{Q}}^{T}\right) \right\} =\text {rank}\left\{ {\varvec{G}}\left( {\varvec{Q}}{\varvec{Q}}^{T}\right) \right\} =\text {rank}\left\{ {\varvec{Q}}{\varvec{Q}}^{T}\right\} , \end{aligned}$$

where the second equality holds because of Corollary 9.3.8 in Harville (1997). Note that, as the Moore–Penrose inverse,

$$\begin{aligned} \text {rank}\left\{ \left( {\varvec{Q}}{\varvec{Q}}^{T}\right) {\varvec{G}}\left( {\varvec{Q}}{\varvec{Q}}^{T}\right) \right\} =&\text {rank}\left\{ {\varvec{Q}}{\varvec{Q}}^{T}\right\} . \end{aligned}$$

Hence, condition (30) is also satisfied. Further, as a gram matrix,

$$\begin{aligned} \text {rank}\left\{ {\varvec{Q}}{\varvec{Q}}^{T}\right\} =\text {rank}\left\{ {\varvec{Q}}\right\} . \end{aligned}$$

Recall that ${\varvec{Q}}$ is idempotent, then the degrees of freedom can be simplified to

$$\begin{aligned} \text {tr}\left\{ {\varvec{G}}\left( {\varvec{Q}}{\varvec{Q}}^{T}\right) \right\} =&\text {tr}\left\{ {\varvec{Q}}\right\} =L_{j}-K_{j}. \end{aligned}$$

That is, *F* is asymptotically Chi-square distributed with $L_{j}-K_{j}$ degrees of freedom. $\square$

### Proof of Theorem 2

Equation (18) indicates that

$$\begin{aligned} \sqrt{n}{\hat{\varphi }}_{j}^{-1}{\varvec{S}}_{vv,j}^{-1/2}\left( {\varvec{S}}_{vu,j}-{\varvec{S}}_{vz,j}\hat{{\varvec{\theta }}}^{(j)}\right) =&\sqrt{n}{\hat{\varphi }}_{j}^{-1}{\varvec{S}}_{vv,j}^{-1/2}\hat{{\varvec{\Omega }}}^{1/2}\hat{{\varvec{Q}}}\hat{{\varvec{\Omega }}}^{-1/2}\left( {\varvec{S}}_{vy,j}-{\varvec{S}}_{vz,j}{\varvec{\theta }}^{(j)}\right) . \end{aligned}$$

Hence, the quadratic form becomes

$$\begin{aligned}&n\left( {\varvec{S}}_{vy,j}-{\varvec{S}}_{vz,j}\hat{{\varvec{\theta }}}^{(j)}\right) ^{T}\left( {\hat{\varphi }}_{j}^{-2}{\varvec{S}}_{vv,j}^{-1}\right) \left( {\varvec{S}}_{vy,j}-{\varvec{S}}_{vz,j}\hat{{\varvec{\theta }}}^{(j)}\right) \\&\quad = n\left[ \hat{{\varvec{\Omega }}}^{-1/2}\left( {\varvec{S}}_{vy,j}-{\varvec{S}}_{vz,j}{\varvec{\theta }}^{(j)}\right) \right] ^{T}\left( {\hat{\varphi }}_{j}^{-2}\hat{{\varvec{Q}}}^{T}\hat{{\varvec{\Omega }}}^{1/2}{\varvec{S}}_{vv,j}^{-1}\hat{{\varvec{\Omega }}}^{1/2}\hat{{\varvec{Q}}}\right) \left[ \hat{{\varvec{\Omega }}}^{-1/2}\left( {\varvec{S}}_{vy,j}-{\varvec{S}}_{vz,j}{\varvec{\theta }}^{(j)}\right) \right] , \end{aligned}$$

and its weight matrix is symmetric. Hence, the distribution (17) indicates that it converges in distribution to a weighted sum of independent Chi-square random variables with 1 degrees of freedom each and the weights are the eigenvalues of $\varphi _{j}^{-2}{\varvec{Q}}^{T}{\varvec{\Omega }}^{1/2}{\varvec{\Sigma }}_{vv,j}^{-1}{\varvec{\Omega }}^{1/2}{\varvec{Q}}$. Finally, plugging in the expression of ${\varvec{Q}}$ into the weights yields the expression of ${\varvec{\Pi }}$ in the theorem. $\square$

### Proof of Corollary 1

For any ${\varvec{\sigma }}$ values, the expression of ${\varvec{\Pi }}_{j}$ directly indicates

$$\begin{aligned} \varphi _{j}^{2}\text {tr}\left\{ {\varvec{\Pi }}_{j}\right\} =\text {tr}\left\{ {\varvec{\Sigma }}_{vv,j}^{-1}{\varvec{P}}_{j}\left( {\varvec{\sigma }}\right) {\varvec{\Omega }}_{j}\right\}&\text { and }&\varphi _{j}^{4}\text {tr}\left\{ {\varvec{\Pi }}_{j}^{2}\right\} =\text {tr}\left\{ \left( {\varvec{\Sigma }}_{vv,j}^{-1}{\varvec{P}}_{j}\left( {\varvec{\sigma }}\right) {\varvec{\Omega }}_{j}\right) ^{2}\right\} , \end{aligned}$$

where ${\varvec{P}}_{j}\left( {\varvec{\sigma }}\right) ={\varvec{I}}-{\varvec{\Sigma }}_{vz,j}\left( {\varvec{\Sigma }}_{vz,j}^{T}{\varvec{\Sigma }}_{vv,j}^{-1}{\varvec{\Sigma }}_{vz,j}\right) ^{-1}{\varvec{\Sigma }}_{vz,j}^{T}{\varvec{\Sigma }}_{vv,j}^{-1}$. The chain rule yields

31 $$\begin{aligned} \frac{\partial {\varvec{h}}_{j}\left( {\varvec{\sigma }}\right) }{\partial \sigma _{i}}= & {} {\varvec{P}}_{j}\left( {\varvec{\sigma }}\right) \left[ \frac{\partial {\varvec{\Sigma }}_{vy,j}}{\partial \sigma _{i}}-\frac{\partial {\varvec{\Sigma }}_{vz,j}}{\partial \sigma _{i}}{\varvec{\gamma }}_{j}\left( {\varvec{\sigma }}\right) \right] \nonumber \\&-{\varvec{\Sigma }}_{vz,j}\left( {\varvec{\Sigma }}_{vz,j}^{T}{\varvec{\Sigma }}_{vv,j}^{-1}{\varvec{\Sigma }}_{vz,j}\right) ^{-1}\frac{\partial {\varvec{\Sigma }}_{vz,j}^{T}{\varvec{\Sigma }}_{vv,j}^{-1}}{\partial \sigma _{i}}{\varvec{h}}_{j}\left( {\varvec{\sigma }}\right) , \end{aligned}$$

where $\sigma _{i}$ is the *i*th entry in ${\varvec{\sigma }}$. If the model is correctly specified and all MIIVs are valid, then ${\varvec{\gamma }}_{j}\left( {\varvec{\sigma }}\right) ={\varvec{\theta }}_{1}^{(j)}$ and ${\varvec{h}}_{j}\left( {\varvec{\sigma }}\right) ={\varvec{0}}$. Hence, we obtain

$$\begin{aligned} \frac{\partial {\varvec{h}}_{j}\left( {\varvec{\sigma }}\right) }{\partial {\varvec{\sigma }}^{T}}= & {} {\varvec{P}}_{j}\left( {\varvec{\sigma }}\right) \frac{\partial {\varvec{g}}\left( {\varvec{\sigma }}\right) }{\partial {\varvec{\sigma }}^{T}}. \end{aligned}$$

Consequently,

$$\begin{aligned} \varphi _{j}^{2}\text {tr}\left\{ {\varvec{\Delta }}_{j}\right\}= & {} \text {tr}\left\{ {\varvec{\Sigma }}_{vv,j}^{-1}{\varvec{P}}_{j}\left( {\varvec{\sigma }}\right) {\varvec{\Omega }}_{j}{\varvec{P}}_{j}^{T}\left( {\varvec{\sigma }}\right) \right\} \\= & {} \text {tr}\left\{ {\varvec{\Sigma }}_{vv,j}^{-1}{\varvec{P}}_{j}\left( {\varvec{\sigma }}\right) {\varvec{\Omega }}_{j}\right\} ,\\ \varphi _{j}^{4}\text {tr}\left\{ {\varvec{\Delta }}_{j}^{2}\right\}= & {} \text {tr}\left\{ {\varvec{\Sigma }}_{vv,j}^{-1}{\varvec{P}}_{j}\left( {\varvec{\sigma }}\right) {\varvec{\Omega }}_{j}{\varvec{P}}_{j}^{T}\left( {\varvec{\sigma }}\right) {\varvec{\Sigma }}_{vv,j}^{-1}{\varvec{P}}_{j}\left( {\varvec{\sigma }}\right) {\varvec{\Omega }}_{j}{\varvec{P}}_{j}^{T}\left( {\varvec{\sigma }}\right) \right\} \\= & {} \text {tr}\left\{ {\varvec{\Sigma }}_{vv,j}^{-1}{\varvec{P}}_{j}\left( {\varvec{\sigma }}\right) {\varvec{\Omega }}_{j}{\varvec{\Sigma }}_{vv,j}^{-1}{\varvec{P}}_{j}\left( {\varvec{\sigma }}\right) {\varvec{\Omega }}_{j}\right\} , \end{aligned}$$

where the last equality holds since ${\varvec{P}}_{j}^{T}\left( {\varvec{\sigma }}\right) {\varvec{\Sigma }}_{vv,j}^{-1}{\varvec{P}}_{j}\left( {\varvec{\sigma }}\right) ={\varvec{\Sigma }}_{vv,j}^{-1}{\varvec{P}}_{j}\left( {\varvec{\sigma }}\right)$. Therefore, $\text {tr}\left\{ {\varvec{\Pi }}_{j}\right\} =\text {tr}\left\{ {\varvec{\Delta }}_{j}\right\}$ and $\text {tr}\left\{ {\varvec{\Pi }}_{j}^{2}\right\} =\text {tr}\left\{ {\varvec{\Delta }}_{j}^{2}\right\}$.

On the other hand, if the population ${\varvec{\Sigma }}$ is estimated by ${\varvec{S}}$ and ${\varvec{\theta }}_{1}^{(j)}$ by $\hat{{\varvec{\theta }}}_{1}^{(j)}$, then ${\varvec{h}}_{j}\left( {\varvec{s}}\right)$ is not necessarily ${\varvec{0}}$. From equation 31, the difference between $\text {tr}\left\{ \hat{{\varvec{\Delta }}}\right\}$ and $\text {tr}\left\{ \hat{{\varvec{\Pi }}}\right\}$ and the difference between $\text {tr}\left\{ \hat{{\varvec{\Delta }}}^{2}\right\}$ and $\text {tr}\left\{ \hat{{\varvec{\Pi }}}^{2}\right\}$ depends on ${\varvec{h}}_{j}\left( {\varvec{s}}\right)$. $\square$

## Footnotes

1 Below their eq. (31), Fisher and Bollen (2020) list the weight matrix as equal to the inverse of the asymptotic covariance matrix of the polychoric covariance matrix. This is a typographical error and the weight matrix should be ${\varvec{W=I}}$, making it the ULS estimator (private communication with authors).

2 The R package can be found in the online supporting documents. It will also be integrated to the MIIVsem package in the future.
