# Supplementary figures and images for: A unified model-implied instrumental variable approach for structural equation modeling with mixed variables
Source: Psychometrika. 2021 Jun 7;86(2):564–94. doi: 10.1007/s11336-021-09771-4 (PMC8313478; doi:10.1007/s11336-021-09771-4)

Average of absolute relative bias

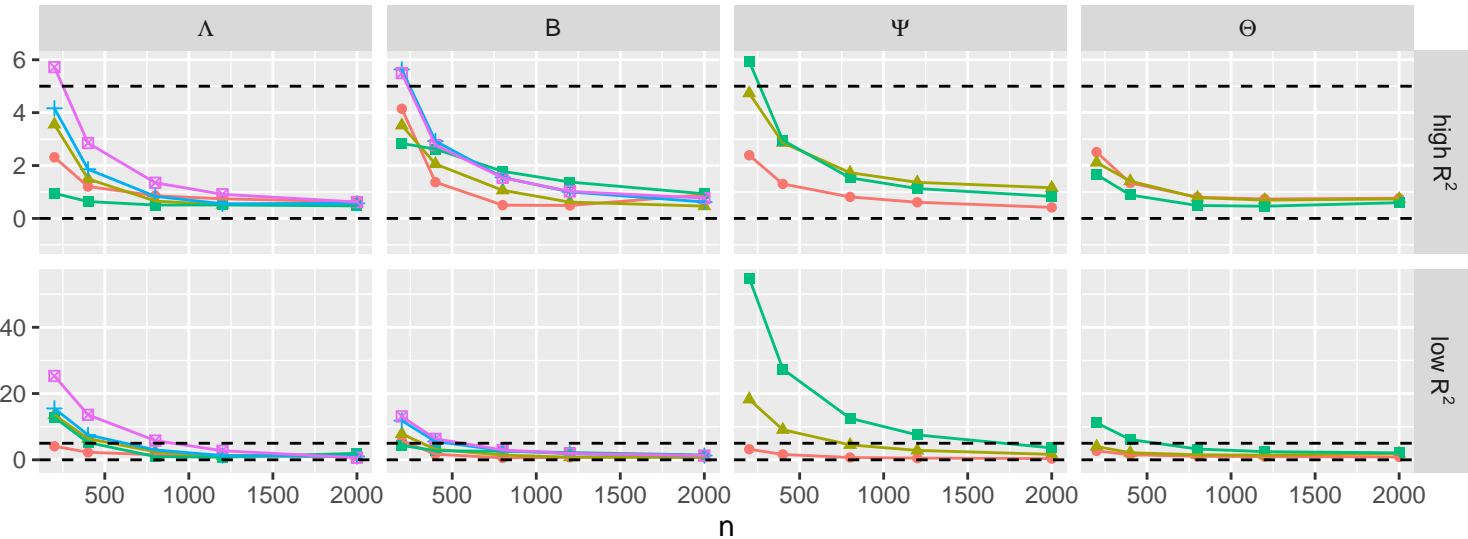

Supplement: Supplementary file 1 — Supplementary material 1 (gz 37 KB) [file 11336_2021_9771_MOESM1_ESM.gz › MIIVmixtype/RBSE(Median)(Correct)(ParSet).pdf]

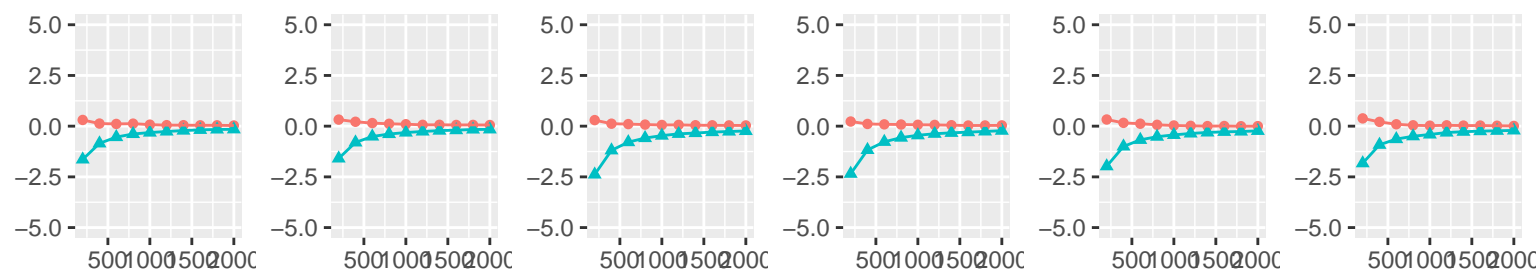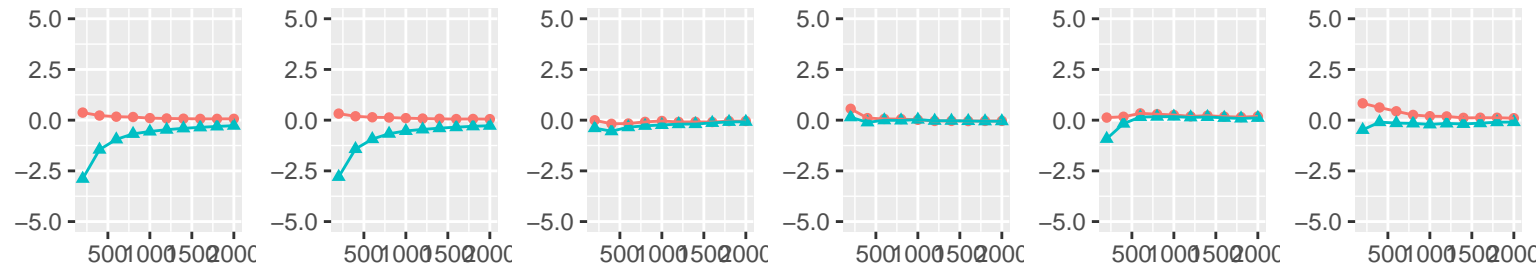

Supplement: Supplementary file 1 — Supplementary material 1 (gz 37 KB) [file 11336_2021_9771_MOESM1_ESM.gz › MIIVmixtype/RBTrial]
